# TRIM27-controlled endothelium-derived exosomes play a central role in podocyte injury in diabetic kidney disease

**DOI:** 10.1038/s41420-026-02953-y

**Published:** 2026-03-07

**Authors:** Yuexin Tian, Yunhe Liu, Xiaojuan Feng, Lunbi Wu, Weiwei Song, Tongyu Zhao, Jinxi Liu, Xinyan Miao, Haimin Ma, Baiyun Jia, Lihua Kang, Qingjuan Liu, Wei Zhang, Huifang Guo, Lin Yang, Jinsheng Xu, Shuxia Liu

**Affiliations:** 1 Department of Pathology, Hebei Key Laboratory of Nephrology, Center of Metabolic Diseases and Cancer Research, Institute of Medical and Health Science, Hebei Medical University, Shijiazhuang, China; 2 Department of Rheumatology, the Second Hospital of Hebei Medical University, Shijiazhuang, China; 3 Department of Nephrology, the Second Hospital of Hebei Medical University, Shijiazhuang, China; 4 Department of Nephrology, Hebei Key Laboratory of Vascular Calcification in Kidney Disease, Hebei Clinical Research Center for Chronic Kidney Disease, the Fourth Hospital of Hebei Medical University, Shijiazhuang, China

**Keywords:** Mechanisms of disease, Diabetic nephropathy, Predictive markers

## Abstract

Exosomes are extracellular vesicles involved in mediating cell–cell communication by shuttling genetic information and proteins. Here, we investigated whether glomerular endothelial cells-derived exosomes play a central role in mediating podocyte injury and proteinuria formation in diabetic kidney disease and its precise mechanism. In vitro, upon stimulation with high glucose and transforming growth factor-β1, primary human renal glomerular endothelial cells (HRGECs) produced more exosomes that directly mediated podocyte injury. Conversely, pharmacological inhibition of exosome secretion by GW4869, knockdown of tripartite motif-containing 27 (TRIM27) expression, or inhibition of miR-486-5p all abolished the ability of high glucose and transforming growth factor-β1-treated HRGECs to induce podocyte injury. In vivo, injections of HRGEC-derived exosomes aggravated podocyte injury and proteinuria in diabetic mice, which was negated by a miR-486-5p inhibitor. Furthermore, specific knockdown of TRIM27 expression or miR-486-5p in endothelial cells preserved kidney function and attenuated podocyte injury in diabetic mice. Thus, our results suggest that TRIM27-induced glomerular endothelial cell-derived exosomes play a major role in podocyte injury by shuttling miR-486-5p in diabetic kidney disease. Hence, strategies targeting exosomes may be a new direction in developing therapeutics for podocyte injury and proteinuria in diabetic kidney disease.

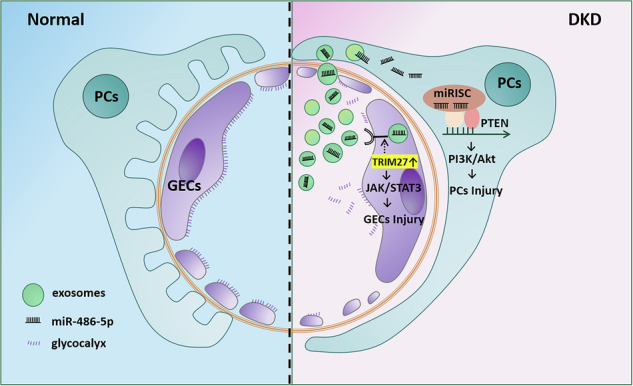

## Introduction

Diabetic kidney disease (DKD) is a major cause of morbidity and mortality in patients with diabetes mellitus and the leading cause of end-stage renal disease. The most characteristic feature of DKD is proteinuria, which is associated with renal progression and cardiovascular events [1]. However, the mechanisms of proteinuria require further exploration.

Podocyte injury is an early event in DKD and significantly contributes to the formation of proteinuria [2]. Recently, crosstalk between endothelial cells (ECs) and podocytes was found to play potential major roles in both repair/regeneration and disease progression [3, 4]. Additionally, podocytes–ECs crosstalk occurs through certain media or information transmission, thereby mediating or aggravating damage to the other, and participating in the occurrence and progression of kidney disease. In diabetic mice, endothelial cell damage caused by a decrease in nitric oxide synthase expression in endothelial cells mediates podocytes damage and severe proteinuria [5]. Increased mtROS and mitochondrial dysfunction are closely related to the damage of endothelial cells and podocytes in diabetes [6]. Angiotensin-2 is upregulated in diabetic nephropathy, and podocyte-specific angiotensin-2 induces endothelial cell apoptosis [7], suggesting that the interaction between endothelial cells and podocytes mediated filtration membrane damage that plays a major role in the formation of proteinuria.

Exosomes are nanoscale vesicles secreted by various cells, which are involved in intercellular communication and material transport by directing the actions of signaling molecules on the surface of the cell membrane and mediating cell contents during membrane fusion [8]. Commonly, exosomes carry proteins, microRNAs (miRNAs) and messenger RNAs (mRNAs), and can be transported between cells. Exosomes abnormality is related to the occurrence and progression of cancer, infectious diseases and neurodegenerative diseases, which is considered to be a potential new target for diagnosis and treatment [9, 10]. In the kidney, almost all intrinsic cells, such as endothelial cells, podocytes, and renal tubular epithelial cells, produce and secrete extracellular vesicles, which are abnormally synthesized and released in kidney diseases such as lupus nephritis and diabetic kidney disease. This occurs in kidney transplantation as well [11, 12]. Whether exosomes are involved in the crosstalk between endothelial cells and podocytes is still unclear.

Tripartite motif-containing 27 (TRIM27) as a DNA-binding protein is a member of the TRIM family, and contains three conserved domains: a RING finger, one or two B-box motifs, and a coiled-coil region. Recently, TRIM27 was found to accelerate the progression of various cancers, such as esophagus, lung, and renal cancers, and involved in the regulation of many cellular functions and biological processes [13, 14]. TRIM27 can up-regulate JAK/STAT signaling pathway to promoting myeloid bias differentiation of hematopoietic stem cells and achieve competitive hematopoiesis formation [15]. We have previously reported that TRIM27 overexpression in the glomeruli of patients or mice with lupus nephritis contributed to endothelial cell injury accompanied by proteinuria. Knocking down the expression of TRIM27 in the kidneys of mice with lupus nephritis can significantly alleviate proteinuria and renal injury [16]. In addition, the overexpression of TRIM27 was observed not only in endothelial cells of patients with lupus nephritis, but also in patients with DKD. However, whether TRIM27 in endothelial cells is related to podocytes injury and contributes to proteinuria and its precise mechanism remains unclear.

In this study, we demonstrate that TRIM27-mediated GEC injury triggers an increase in exosomes of glomerular endothelial cells, which capsulating miR-486-5p. These exosomes mediate intercellular communication by shuttling miR-486-5p from glomerular endothelial cells to podocytes. Our results underlie the importance of exosomes in the pathogenesis of DKD and suggest a novel avenue in developing therapeutics for DKD.

## Results

### TRIM27 mediates GEC injury by activating the JAK2/STAT3 signal pathway in DKD

**Fig. 1:**
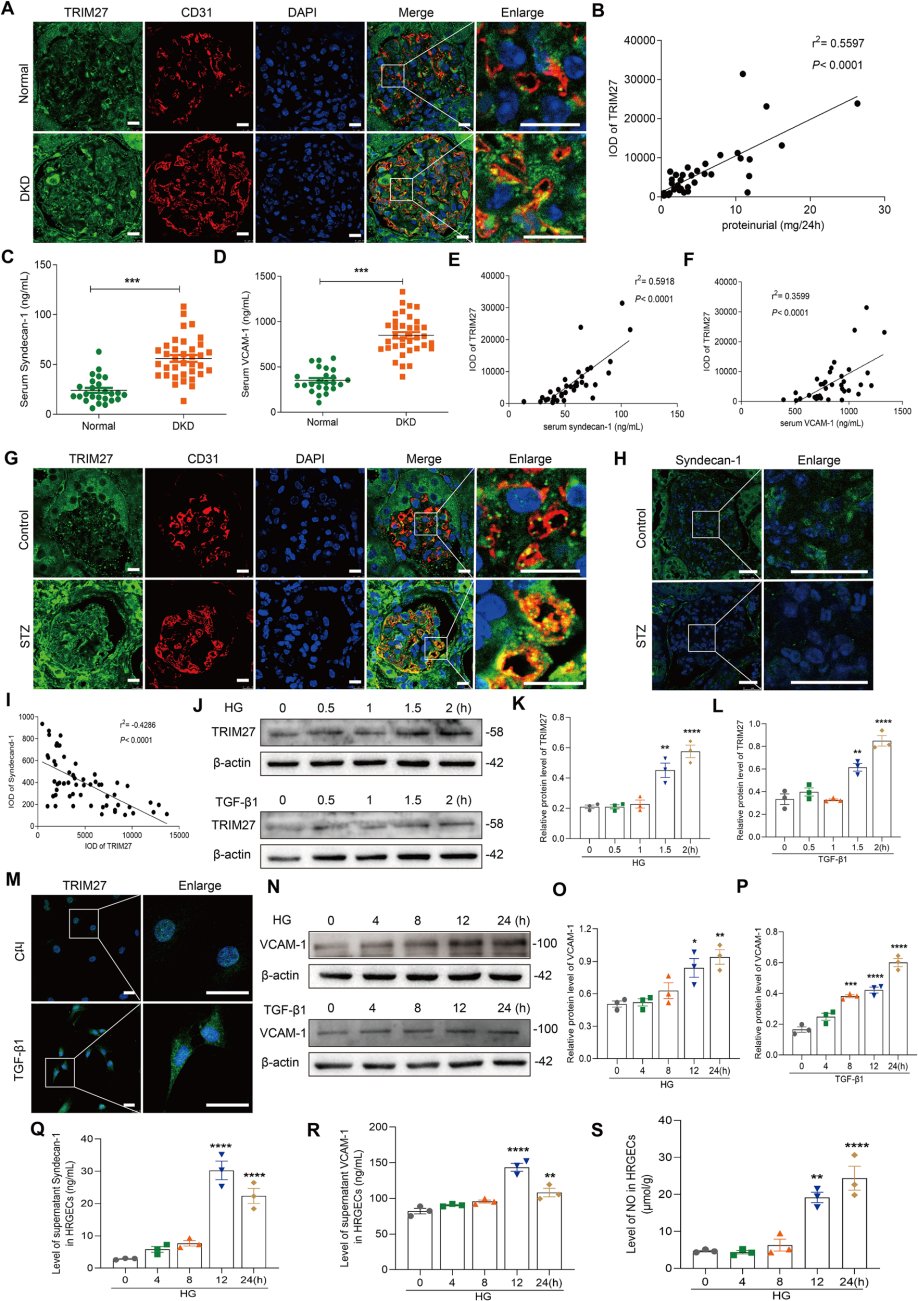
TRIM27 increases in GECs and positively correlates with proteinuria and EC injury in DKD. **A** Double immunofluorescence staining of TRIM27 protein in glomerular endothelial cells of DKD patients. Kidney sections were costained for TRIM27 (green) and specific endothelial cell marker CD31 (red). Scale bars: 10 μm. **B** Significant positive correlation was observed between TRIM27 expression and proteinuria in DKD patients (*n* = 36 DKD patients). **C**, **D** The ELISA assays showed serum contents of syndecan-1 and VCAM-1 increased in DKD patients. ^***^P < 0.001 vs. Normal group (*n* = 24 normal controls and 36 DKD patients). **E**, **F** Significant positive correlation was observed between TRIM27 expression and syndecan-1 and VCAM-1 serum levels in DKD patients (*n* = 36 DKD patients). **G** Expression of TRIM27 (green) in glomerular endothelial cells (specific marker, CD31; red) of STZ-induced diabetec mice detected by immunofluorescence. Scale bars: 10 μm. **H** Expression of syndecan-1 in the glomerulus of STZ mice detected by immunofluorescence. Scale bars: 25 μm. **I** Significant negative correlation was observed between TRIM27 and syndecan-1 expression in STZ mice (*n* = 6 mice, 10 glomeruli per mouse). **J**–**L** Western blot assay showed TRIM27 expression increased in HRGECs treated with HG or TGF-β1. ^**^P < 0.01, ^****^P < 0.0001 vs. 0 h group (*n* = 3). **M** IF assay showed that the expression of TRIM27 increased in HRGECs treated with TGF-β1. Scale bars: 25 μm. **N**–**P** Western blot assay showed VCAM-1 expression increased in HRGECs treated with HG or TGF-β1. ^*^P < 0.05, ^**^P < 0.01, ^***^P < 0.001, ^****^P < 0.0001 vs. 0 h group (*n* = 3). **Q**, **R** The ELISA assays showed the contents of syndecan-1 and VCAM-1 increased in cultures supernatant of HRGECs treated with HG for 24 h. ^**^P < 0.01, ^****^P < 0.0001 vs. 0 h group (*n* = 3). **S** NO level increased in the HRGECs after treated with HG. ^**^P < 0.01, ^****^P < 0.0001 vs. 0 h group (*n* = 3). Student’s *t* test and Bonferroni’s correction were performed to analyze statistical significance. Pearson’s method was used for correlation analysis. Data are the mean ± SEM.

To determine the role of TRIM27 in DKD, we measured TRIM27 expression in glomerular endothelial cells (GECs) of DKD patients and streptozotocin (STZ)-induced diabetic mice. As shown in Fig. 1A, the TRIM27 expression was co localized with CD31, a marker of endothelial cells, and TRIM27 expression was increased in GECs of the DKD group compared with the control group (Supplementary Fig. S1B). More importantly, the IOD of TRIM27 in glomerular endothelial cells of DKD patients showed a significant positive correlation to proteinuria (Fig. 1B). Additionally, there was a significant positive correlation between TRIM27 and serum syndecan-1 or VCAM-1 of DKD patients, which all significantly increased in the serum of DKD patients (Fig. 1C–F). Similarly, TRIM27 was co localized with CD31 and increased in GECs of STZ mice following a decrease in syndecan-1 expression in glomerular endothelial cells (Fig. 1G, H and Supplementary Fig. S1C, D). TRIM27 showed a significant negative correlation to syndecan-1 in glomeruli (Fig. 1I).

**Fig. 2:**
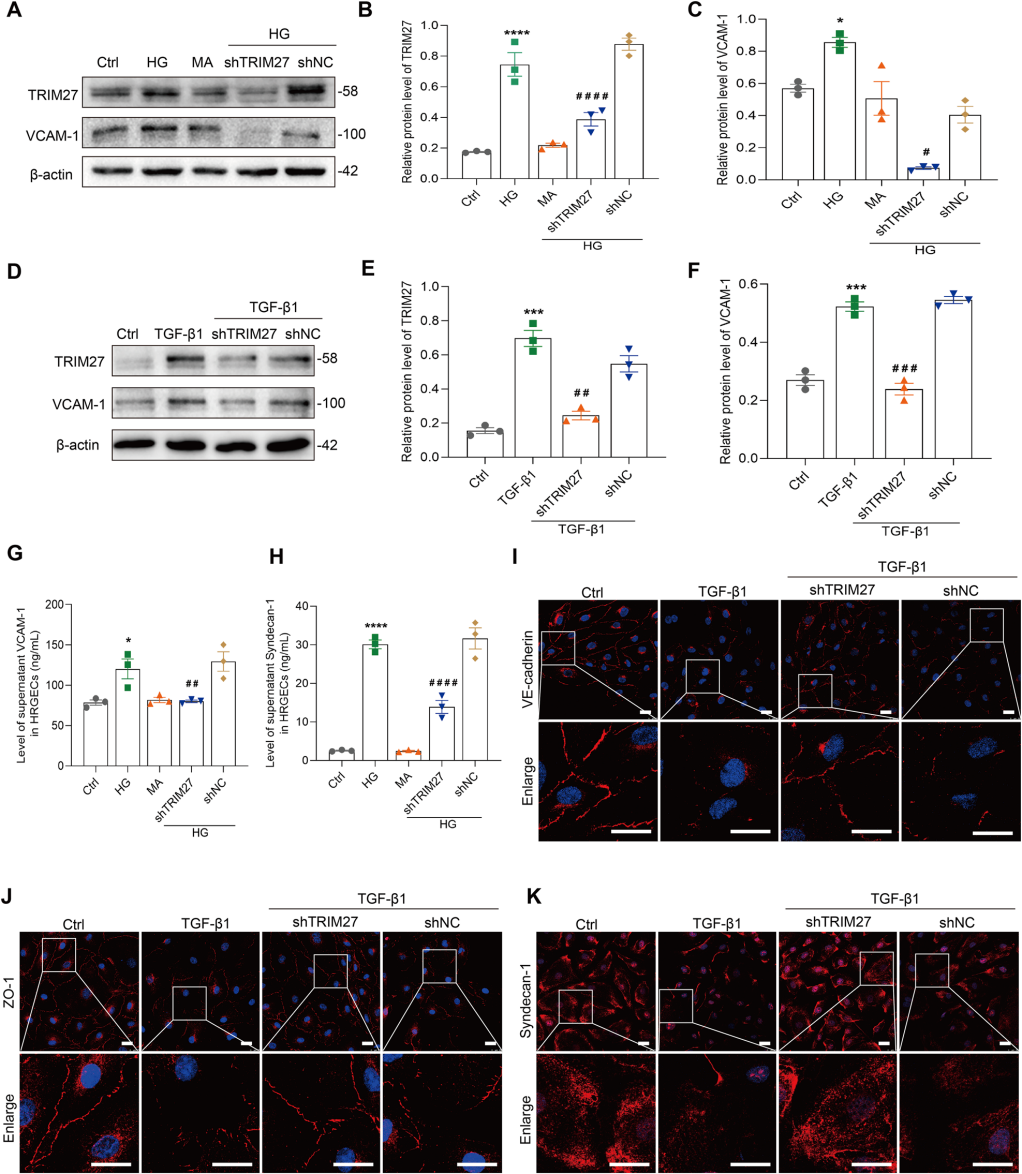
TRIM27 regulates HRGEC injury induced by HG or TGF-β1. **A**–**F** Western blot assay showed TRIM27 and VCAM-1 expression decreased in HRGECs treated with shTRIM27 and HG or TGF-β1 for 24 h. ^*^P < 0.05, ^***^P < 0.001, ^****^P < 0.0001 vs. control group, ^#^*P* < 0.05, ^####^*P* < 0.0001 vs. HG+shNC group, ^##^*P* < 0.01, ^###^*P* < 0.001 vs. TGF-β1+shNC group (*n* = 3). **G**, **H** ELISA assays showed contents of syndecan-1 and VCAM-1 decreased in culture supernatants after TRIM27 knockdown in HRGECs and treatment with HG for 24 h. ^*^P < 0.05, ^****^P < 0.0001 vs. control group, ^##^*P* < 0.01, ^####^*P* < 0.0001 vs. HG+shNC group (*n* = 3). **I**–**K** IF assay showed that downregulation of TRIM27 increased VE-cadherin, ZO-1, and syndecan-1 expression in HRGECs treated with TGF-β1 for 24 h. Scale bars: 25 μm. Bonferroni’s correction was performed to analyze statistical significance. Data are the mean ± SEM.

To further explore the precise mechanism of TRIM27-induced GEC injury, in vitro experiments were performed. Both HG and TGF-β1 increased TRIM27 expression in a time-dependent manner and induced HRGEC injury with upregulation of VCAM-1 and NO, and syndecan-1 shedding (Fig. 1J–S and Supplementary Fig. S1E). Knockdown of TRIM27 decreased VCAM-1 expression, improved the skeletal structure, and relieved glycocalyx shedding of endothelial cells exposed to HG or TGF-β1 (Fig. 2A–K and Supplementary Fig. S1F–H).

**Fig. 3:**
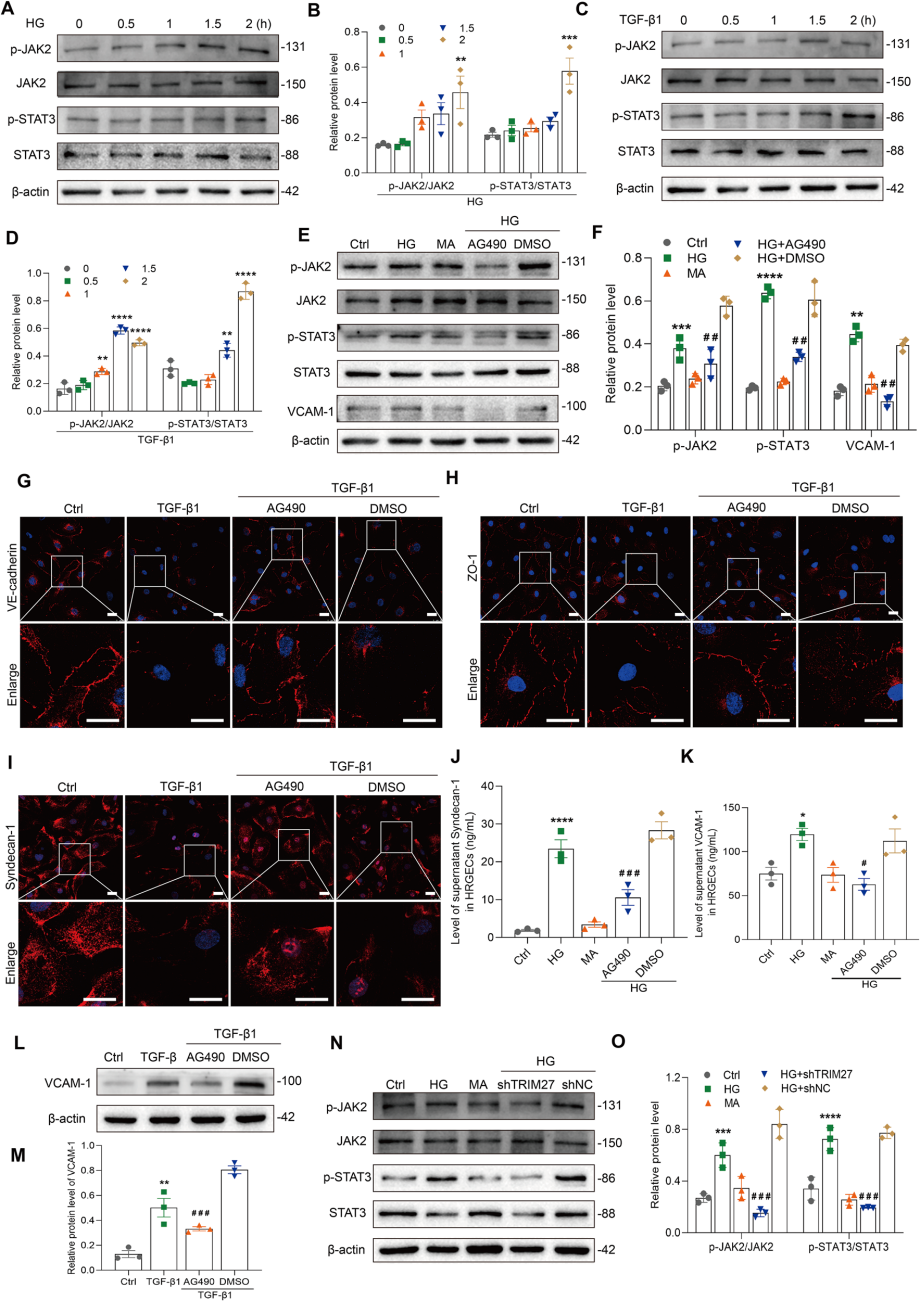
TRIM27 regulates HRGEC injury caused by HG or TGF-β1 by activating the JAK2/STAT3 signal pathway. **A**–**D** Western blot assay showed p-JAK2 (Y1007) and p-STAT3 (Tyr705) expression increased in HRGECs treated with HG or TGF-β1. ^**^P < 0.01, ^***^P < 0.001, ^****^P < 0.0001 vs. 0 h group (*n* = 3). **E**, **F** Western blot assay showed p-JAK2 (Y1007), p-STAT3 (Tyr705), and VCAM-1 expression decreased after inhibition of the JAK2/STAT3 pathway by AG490 in HRGECs and treatment with HG for 24 h. ^**^P < 0.01, ^***^P < 0.001, ^****^P < 0.0001 vs. control group, ^##^*P* < 0.01 vs. HG + DMSO group (*n* = 3). **G**–**I** IF assay showed that VE-cadherin, ZO-1, and syndecan-1 expression increased in HRGECs treated with AG490 and TGF-β1 for 24 h. Scale bars: 25 μm. **J**, **K** The ELISA assays showed contents of syndecan-1 and VCAM-1 in culture supernatants of HRGECs decreased after treated with AG490 and HG for 24 h. ^*^P < 0.05, ^****^P < 0.0001 vs. control group, ^#^*P* < 0.05, ^###^*P* < 0.001 vs. HG + DMSO group (*n* = 3). **L**, **M** Western blot showed that VCAM-1 expression decreased after inhibition of the JAK2/STAT3 pathway in HRGECs. ^**^P < 0.01 vs. control group, ^###^*P* < 0.001 vs. TGF-β1 + DMSO group (*n* = 3). **N**, **O** Western blot assay showed downregulation of TRIM27 decreased p-JAK2 (Y1007) and p-STAT3 (Tyr705) expression in HRGECs treated with HG for 2 h. ^***^P < 0.001, ^****^P < 0.0001 vs. control group, ^###^*P* < 0.001 vs. HG+shNC group (*n* = 3). Bonferroni’s correction was performed to analyze statistical significance. Data are the mean ± SEM.

To further elucidate the mechanism of TRIM27-mediated injury of GECs in DKD, we conducted proteomic screening on HRGECs subjected to HG stimulation. Proteomic analysis revealed a significant down-regulation of STAT3 expression in HRGECs following knockdown of TRIM27 (Supplementary Fig. S2A). Previous studies by Zheng et al. have effectively demonstrated the regulatory relationship between TRIM27 and JAK/STAT signaling pathway in their investigation into the role of TRIM27 in activating STAT3 to promote colitis,colitis-associated carcinogenesis and hepatocellular carcinoma [17, 18]. Their research indicates that TRIM27 is crucial for the formation of the JAK-STAT3 complex. In order to gain further insight into the underlying mechanism of TRIM27-mediated injury to GECs in DKD, we analyzed the JAK2/STAT3 signaling pathway. Figure 3A–D show that p-JAK2 (Y1007) and p-STAT3 (Tyr705) expression was increased in HRGECs stimulated by HG or TGF-β1 at 2 h, while AG490, a specific inhibitor of JAK2/STAT3 signaling, improved TGF-β1 and HG-induced injury of HRGECs expressed by decreasing VCAM-1 expression, syndecan-1 shedding, and the expression and arrangement of VE-cadherin and ZO-1 (Fig. 3E–M and Supplementary Fig. S2B–D). However, downregulation of TRIM27 in the HG-induced group inhibited activation of the JAK2/STAT3 pathway (Fig. 3N, O). Under HG and TGF-β1 stimulation, coimmunoprecipitation assays revealed an interaction between TRIM27 and JAK2/STAT3 in HRGECs (Supplementary Fig. S2E).

Therefore, the TRIM27 mediated glomerular endothelial cell injury by activating the JAK2/STAT3 signaling pathway in DKD.

### Diabetic mice with improved endothelial dysfunction induced by knockdown of TRIM27 achieve podocyte injury relief

**Fig. 4:**
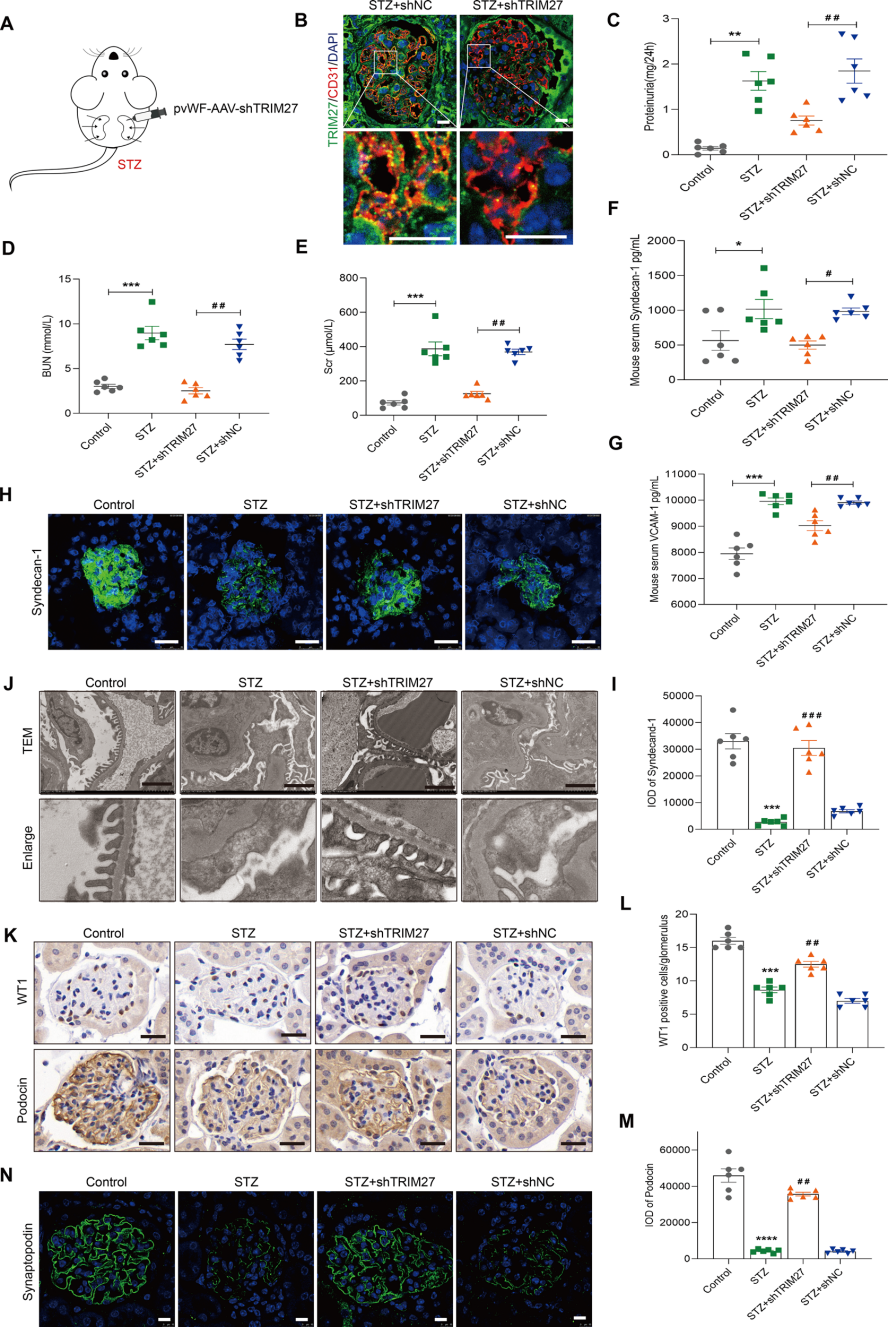
Specific knockdown of TRIM27 expression in GECs suppresses GEC and podocyte injury in STZ mice. **A** Eighteen 20-week-old mice were randomly divided into three groups: STZ (*n* = 6), STZ+shTRIM27 (*n* = 6), and STZ+shNC group (*n* = 6). Mice in STZ+shTRIM27 and STZ+shNC groups were renally injected with 50 μl of 1 × 10^11^ infective units of adeno-associated virus at three sites each in both kidneys. Six control mice and 6 STZ mice were injected with isometric saline. The mice were sacrificed after 4 weeks. **B** IF staining showed TRIM27 expression decreased in glomerular endothelial cells of STZ+shTRIM27 mice. Scale bars: 10 μm. **C**–**E** Level of 24-h proteinuria, BUN, and Scr decreased in STZ+shTRIM27 mice. ^**^P < 0.01, ^***^P < 0.001 vs. Control mice, ^##^*P* < 0.01 vs. STZ+shNC mice. *n* = 6 each group. **F**, **G** The ELISA assays showed serum contents of syndecan-1 and VCAM-1 decreased in STZ+shTRIM27 mice. ^*^P < 0.05, ^***^P < 0.001 vs. Control mice, ^#^*P* < 0.05, ^##^*P* < 0.01 vs. STZ+shNC mice. *n* = 6 each group. **H**, **I** IF staining showed syndecan-1 expression increased in STZ+shTRIM27 mice. Scale bars: 25 μm. ^***^P < 0.001 vs. Control mice, ^###^*P* < 0.001 vs. STZ+shNC mice (*n* = 6). **J** Electron microscopy assay showed ultrastructure of the podocytes in the renal cortex of mice. Scale bars: 2 μm. **K**–**M** IHC staining showed WT1 and podocin expression increased in STZ+shTRIM27 mice. Scale bars: 25 μm. ^***^P < 0.001 vs. Control mice, ^##^*P* < 0.01 vs. STZ+shNC mice (*n* = 6). **N** IF staining showed synaptopodin expression increased in STZ+shTRIM27 mice. Scale bars: 10 μm. Bonferroni’s correction was performed to analyze statistical significance. Data are the mean ± SEM.

To further explore whether TRIM27 mediated GEC injury in vivo, STZ mice with abnormal kidney functions were renally injected with AAV-shTRIM27 with endothelial cell-specific promoter pvWF (Fig. 4A). As shown in Fig. 4B, the expression of TRIM27 was decreased in GECs of the STZ+shTRIM27 mice compared with STZ+shNC mice, while the expression in other cells was unchanged (Supplementary Fig. S3A). Notably, specific knockdown of TRIM27 in ECs alleviated proteinuria, BUN, and Scr in STZ mice (Fig. 4C–E), and improved syndecan-1 and VCAM-1 expression in mouse serum and endothelial cells (Fig. 4F–I). More interestingly, TEM revealed a significant increase in podocyte effacement in STZ mice compared with control mice, but a remarkable reduction in STZ+shTRIM27 mice (Fig. 4J). Additionally, the podocyte number was estimated by podocyte marker WT1. The number of WT1-positive cells per glomerulus was significantly increased in STZ+shTRIM27 mice compared with STZ+shNC mice. Expression of podocyte-specific genes (podocin and synaptopodin) was also increased in STZ+shTRIM27 mice as determined by IHC and IF staining of kidney sections (Fig. 4K–N and Supplementary Fig. S3B). Furthermore, pathological changes were alleviated by TRIM27 knockdown (Supplementary Fig. S3C). Moreover, db/db mice were renally injected with pvWF-AAV-shTRIM27 (Supplementary Fig. S4A), and similar results were observed (Supplementary Fig. S4B–M).

Taken together, specific knockdown of TRIM27 expression in endothelial cells alleviated podocyte injury and proteinuria in DKD along with improved endothelial cell dysfunction, suggesting possible crosstalk between glomerular endothelial cells and podocytes.

### Glomerular endothelial cell-derived exosomes mediate podocyte injury in DKD

**Fig. 5:**
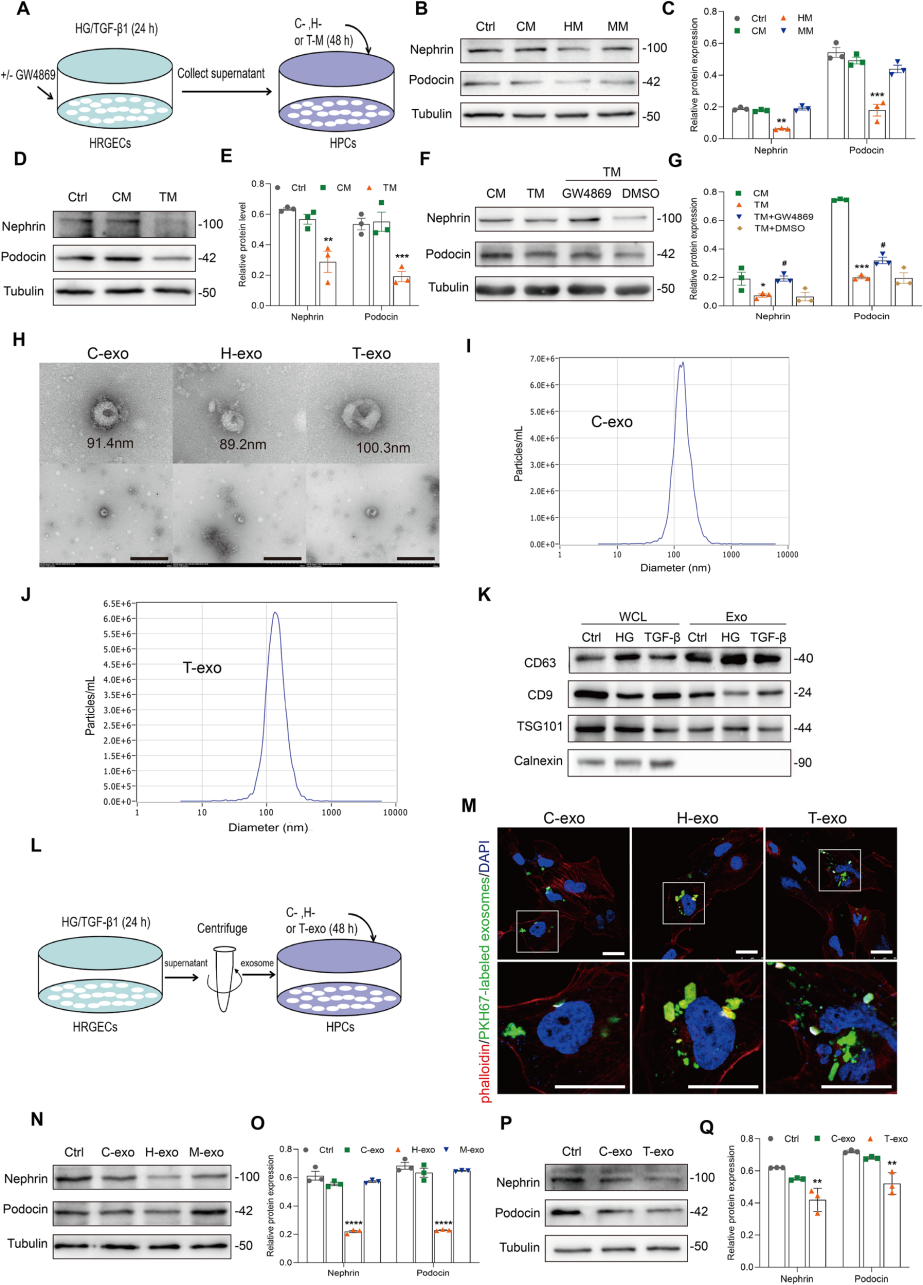
The HRGEC-derived exosomes promote podocyte injury. **A** Experimental design. HRGECs were treated with HG or TGF-β1 for 24 h in the absence or presence of GW4869, an inhibitor exosome biogenesis and release. Stimuli were removed and the cells were incubated for an additional 24 h in control medium. Conditioned media were collected and used to stimulate HPCs. Control conditioned medium (CM); HG conditioned medium (HM); MA conditioned medium (MM); TGF-β1 conditioned medium (TM). **B**–**G** Western blot assay showed nephrin and podocin expression in the HPCs after incubation in HG or TGF-β1 conditioned media for 48 h. ^*^P < 0.05, ^**^P < 0.01, ^***^P < 0.001 vs. CM group, ^#^*P* < 0.05 vs. TM + DMSO group (*n* = 3). **H** TEM showed that exosomes isolated from conditioned media of HRGECs. Scale bars: 500 nm. **I**, **J** Concentrations and size distributions of C-exo and T-exo were determined by NTA. **K** Western blot assay showed the expression of exosome-positive markers (CD63, CD9, and TSG101) and an exosome-negative marker (calnexin) in HRGECs and HRGEC-derived exosomes. **L** Experimental design. Exosomes were isolated from conditioned media. Control exosome (C-exo); HG exosome (H-exo); MA exosome (M-exo); TGF-β1 exo (T-exo). **M** IF confirmed intracellular transfer of HRGEC-derived exosomes in HPCs. Scale bars: 25 μm. **N**–**Q** Western blot assay showed nephrin and podocin expression decreased in HPCs after incubation with HG or TGF-β1 exosomes for 48 h. ^**^P < 0.01, ^****^P < 0.0001 vs. C-exo group (*n* = 3). Bonferroni’s correction was performed to analyze statistical significance between multiple comparisons. Data are the mean ± SEM.

Next, to investigate the potential relationship between glomerular endothelial cells and podocytes in DKD, the conditioned media were prepared as described previously [19] and applied to HPCs for 48 h (Fig. 5A). As shown in Fig. 5B–E, compared with the control conditioned medium group (CM), nephrin and podocin expression was decreased in HPCs of HG-conditioned medium (HM) and TGF-β1-conditioned medium (TM) groups.

Exosomes carrying proteins, microRNAs (miRNAs), and messenger RNAs (mRNAs) are involved in intercellular communication and material transport [20]. To explore whether exosomes mediated the crosstalk between glomerular endotheliocyte and podocyte, GW4869 was used to block the biogenesis and release of exosomes [21, 22]. As shown in Fig. 5F, G, GW4869 improved nephrin and podocin expression, suggesting that exosomes played an important role in glomerular endothelial cell–podocyte crosstalk.

To confirm the role of GEC-derived exosomes in podocyte injury, we isolated exosomes from culture medium of HRGECs treated with or without HG or TGF-β1. TEM revealed intact cup-shaped membrane vesicles with sizes in accordance with the nanoparticle tracking analysis. Furthermore, the immunodetection confirmed positive expression of exosomal markers CD63, CD9 and TSG101, while the organelle marker calnexin was undetectable in these isolated vesicles (Fig. 5H–L). Next, the GEC-derived exosomes were labeled with PKH67, a fluorescent lipophilic dye for long-term tracing, and then incubated with HPCs. As shown in Fig. 5M, the PKH67-labeled exosomes were taken up by HPCs. Additionally, compared with the control exosome (C-exo) group, nephrin and podocin expression was decreased in HPCs of HG exosome (H-exo) and TGF-β1 exo (T-exo) groups (Fig. 5N–Q).

### TRIM27 mediates podocyte injury by upregulating the secretion of GEC-derived exosomes in DKD

**Fig. 6:**
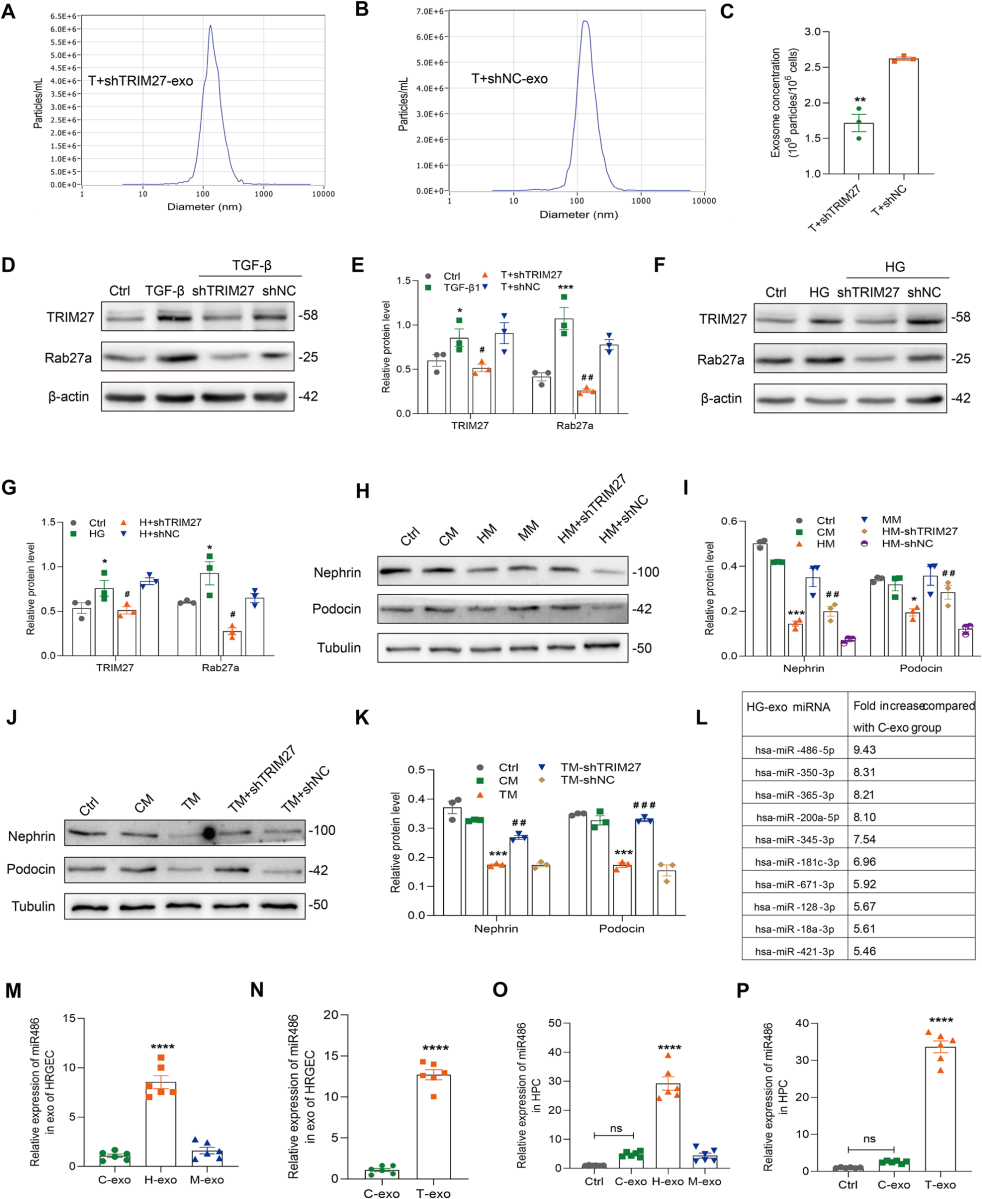
TRIM27 regulates podocyte injury by mediating secretion of GEC-derived exosomes. **A**, **B** Concentrations and size distributions of T+shTRIM27-exo and T+shNC-exo determined by NTA. **C** Quantification of NTA results from three independent experiments. ^**^P < 0.01 vs. T+shTRIM27 group (*n* = 3). **D**–**G** Western blot assay showed downregulation of TRIM27 decreased Rab27a expression in HRGECs treated with TGF-β1 or HG for 24 h. ^*^P < 0.05, ^***^P < 0.001 vs. control group, ^#^*P* < 0.05, ^##^*P* < 0.01 vs. TGF-β1+shNC or HG+shNC group (*n* = 3). **H**–**K** Western blot assay showed conditional media from HRGECs with knocked down TRIM27 increased nephrin and podocin expression in HPCs. ^*^P < 0.05, ^***^P < 0.001 vs. CM group, ^##^*P* < 0.01, ^###^*P* < 0.001 vs. HM+shNC group or TM+shNC group (*n* = 3). **L** Table of the 10 most abundant miRNAs in HG exosomes compared with control exosomes. **M**–**P** Detection of miR-486-5p in HRGEC-derived exosomes and HPCs treated with exosomes by qPCR normalized to U6. ^****^P < 0.0001 vs. C-exo group, ns. no significance (*n* = 6). Student’s *t* test and Bonferroni’s correction were performed to analyze statistical significance. Values are the mean ± SEM.

To further explore the role of TRIM27, HRGECs were transfected with shTRIM27. More importantly, the nanoparticle tracking analysis of the pellets revealed a decrease in the number of particles secreted by HRGECs upon TRIM27 knockdown compared with negative control cells, demonstrating that downregulating TRIM27 caused a decrease in exosomes secretion (Fig. 6A–C). Next, we measured expression of Rab27a, an important protein in exosome synthesis and secretion. As shown in Fig. 6D–G, Rab27a expression was increased in HG and TGF-β1 groups, but decreased upon knockdown of TRIM27 expression. Furthermore, the stability of Rab27a did not differ between the shTRIM27 group and the shNC group (Supplementary Fig. S5A). In contrast, TRIM27 knockdown significantly decreased Rab27a mRNA expression (Supplementary Fig. S5B, C). The JASPAR database (http://jaspar.genereg.net/) was used to predicte and analyze that STAT3 acts as a transcription factor for the Rab27a gene. Additionally, knockdown of STAT3 in HRGECs decreased both Rab27a protein and mRNA levels (Supplementary Fig. S5D–G). Using the JASPAR database to predict STAT3 binding sites in the Rab27a promoter revealed two putative motifs (Supplementary Fig. S5H). To validate these predictions, ChIP-qPCR assays was conducted to confirm STAT3’s binding site in this promoter, and data showed that TF2 (not TF1) is the probable STAT3-binding site (Supplementary Fig. S5I). To further validate the STAT3-TF2 interaction, we designed luciferase reporter plasmids harboring the wild-type (WT2) or mutant (MUT2) TF2 site of the Rab27a promoter and transfected into 293 T cells. Luciferase activity assays revealed that STAT3 overexpression enhanced reporter activity driven by the WT2 Rab27a promoter, whereas no such effect was observed for the MUT2 promoter (Supplementary Fig. S5J, K). Collectively, these data indicate that TRIM27 upregulates Rab27a to mediate exosome synthesis and secretion by promoting the activation of its transcription factor STAT3. Additionally, as shown in Fig. 6H–K, compared with HM+shNC and TM+shNC groups, nephrin and podocin expression was increased in HPCs of HM+shTRIM27 and TM+shTRIM27 groups.

### miR-486-5p in GEC-derived exosomes plays a major role in podocyte injury in DKD

To further explore which components in GEC-derived exosomes were responsible for inducing podocyte injury, gene chip technology was used to screen differentially expressed miRNAs. In HG exosomes, miR-486-5p was 9.43-fold higher than in control exosomes (Fig. 6L). qPCR revealed upregulation of miR-486-5p in HG-Exo and TGF-β1-Exo compared with the control group (Fig. 6M, N). Additionally, HG and TGF-β1 exosomes upregulated miR-486-5p expression in HPCs (Fig. 6O, P).

**Fig. 7:**
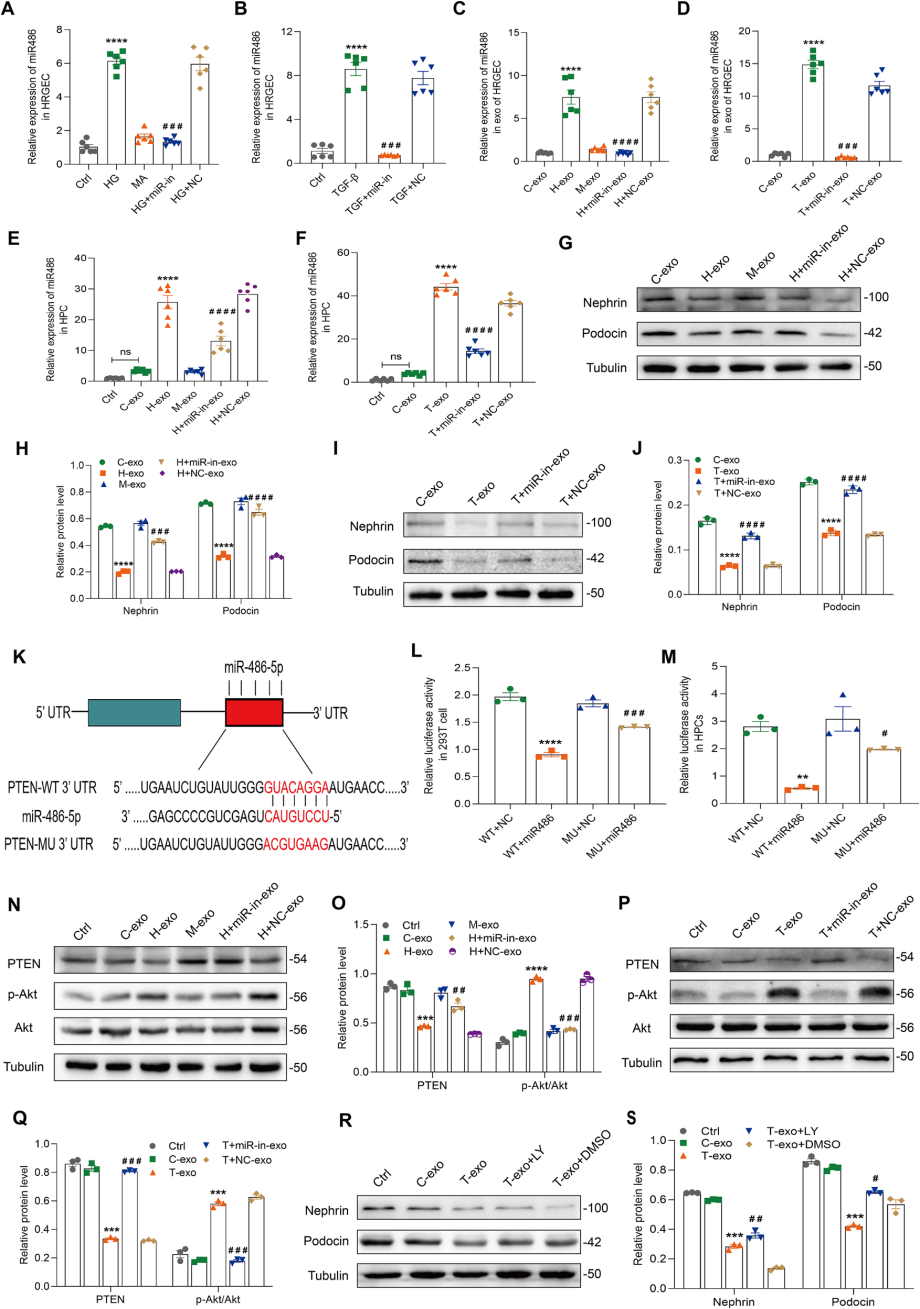
miR-486-5p in GEC-derived exosomes plays a major role in podocyte injury via the PTEN/Akt pathway. **A**, **B** Detection of miR-486-5p in HRGECs after treatment with the miR-486-5p inhibitor by qPCR normalized to U6. ^****^P < 0.0001 vs. control group, ^###^*P* < 0.001 vs. HG+miR-in group or TGF+miR-in group (*n* = 6). **C**, **D** The qPCR assay showed miR-486-5p expression in HRGEC-derived exosomes normalized to U6. ^****^P < 0.0001 vs. C-exo group, ^###^*P* < 0.001, ^####^*P* < 0.0001 vs. H + NC-exo group or T + NC-exo group (*n* = 6). **E**, **F** Detection of miR-486-5p in HPCs treated with exosomes by qPCR normalized to U6. ^****^P < 0.0001 vs. C-exo group, ^####^*P* < 0.0001 vs. HG + NC-exo group or TGF + NC-exo group (*n* = 6). **G**–**J** Western blot assay showed nephrin and podocin expression increased in HPCs treated with exosomes containing a low level of miR-486-5p. ^****^P < 0.0001 vs. C-exo group, ^###^*P* < 0.001, ^####^*P* < 0.0001 vs. H + NC-exo group or T + NC-exo group (*n* = 3). **K** Luciferase reporter assays were used to confirm targeting of the PTEN 3’-untranslated region (UTR) by miR-486-5p. **L**, **M** Dual luciferase reporter assay was performed to confirm the binding site of miR-486-5p and PTEN. ^**^P < 0.01, ^****^P < 0.0001 vs. WT + NC group, ^#^*P* < 0.05, ^###^*P* < 0.001 vs. WT+miR-486 group (*n* = 3). **N**–**Q** Western blot assay showed PTEN and p-AKT (S473) expression in the HPCs treated with exosomes containing a low level of miR-486-5p. ^***^P < 0.001, ^****^P < 0.0001 vs. C-exo group, ^##^*P* < 0.001, ^###^*P* < 0.001 vs. H + NC-exo group or T + NC-exo group (*n* = 3). **R**, **S** Western blot assay showed nephrin and podocin expression increased in HPCs pretreated with LY294002, an inhibitor of Akt, and exposed to TGF-β1 exosomes for 48 h. ^***^P < 0.001 vs. C-exo group, ^#^*P* < 0.05, ^##^*P* < 0.01 vs. T-exo+DMSO group (*n* = 3). Bonferroni’s correction was performed to analyze statistical significance. Data are the mean ± SEM.

Subsequently, to investigate the role of exosomal miR-486-5p in podocyte injury, we downregulated miR-486-5p in HRGECs by a miR-486-5p inhibitor. As shown in Fig. 7A–D, miR-486-5p was all knocked down in HRGECs and HRGEC-derived exosomes. More importantly, the knockdown of miR-486-5p in exosomes downregulated miR-486-5p in podocytes and reversed the decrease of nephrin and podocin in HPCs treated with HG-exo and TGF-β1-exo (Fig. 7E–J). These results confirmed that miR-486-5p encapsulated in GEC-derived exosomes plays a crucial role in mediating podocyte injury in DKD.

A target scan assay and other experiments have shown that PTEN is a possible target gene of miR-486-5p [23, 24]. To explore the possible mechanism of miR-486-5p in inducing podocyte injury, we analyzed PTEN/Akt pathway. As shown in Fig. 7K–M, HEK293T cells and HPCs incubated with miR-486-5p mimic were transfected with the WT-reporter vector, luciferase activity was reduced significantly, and luciferase activity was significantly enhanced after transfection with the MU-reporter vector. Additionally, PTEN expression was reduced in HPCs stimulated with HG or TGF-β1 exosomes and upregulated when miR-486-5p was knocked down. However, the p-Akt (S473) was increased in HPCs treated with HG or TGF-β1 exosomes, which was reversed after knockdown of miR-486-5p (Fig. 7N–Q). Furthermore, we found that PTEN overexpression downregulated the expression of p-Akt (S473) and reverse HPC injury (Supplementary Fig. S6). Additionally, inhibition of Akt activation by LY294002 reversed HPC injury induced by GEC-derived exosomes exposed to TGF-β1 (Fig. 7R, S). Therefore, GEC-derived exosomes transferred miR-486-5p to podocytes, and mediated podocyte injury by targeting the PTEN/Akt pathway.

### The GEC-derived exosomes promote podocyte injury in vivo

**Fig. 8:**
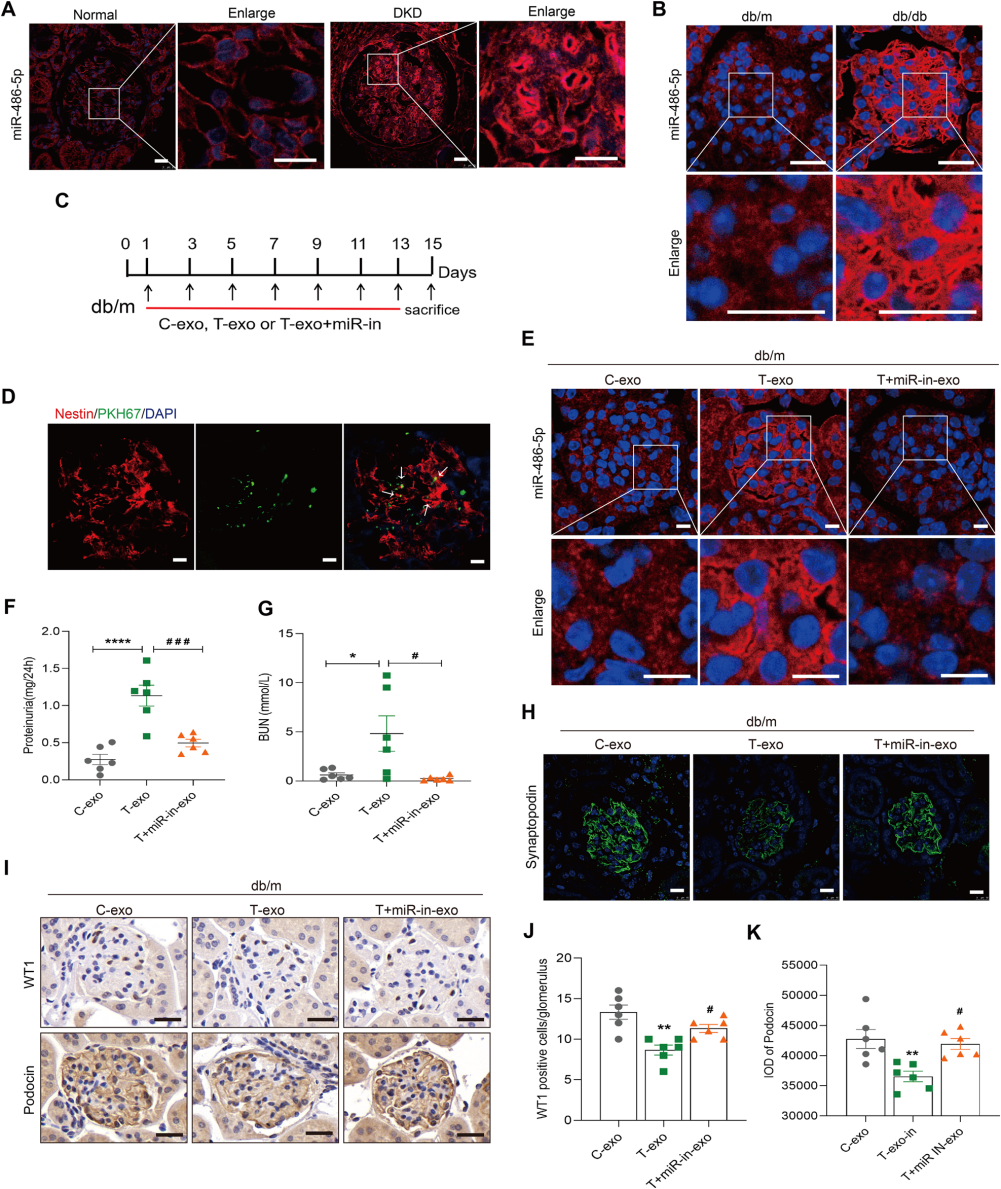
HRGEC-derived exosomes promote podocyte injury in db/m mice. **A**, **B** FISH assay showed miR-486-5p expression and localization in DKD patients and db/db mice. Scale bars: 25 μm. **C** Experimental design. Eighteen 10-week-old mice were randomly divided into three groups: C-exo (*n* = 6), T-exo (*n* = 6), and T-exo+miR-in (*n* = 6). Arrows indicate the time points when HRGEC-derived exosomes were injected i.v. **D** IF showed PKH67-labeled exosomes in mouse kidneys after i.v. injection. Nestin (green) was used as a podocyte-specific marker. White arrowheads indicate costaining of exosomes with nestin. Scale bars: 10 μm. **E** FISH assay showed miR-486-5p expression and localization in mice injected with exosomes. Scale bars: 10 μm. **F**, **G** Levels of 24-h proteinuria and BUN in mice. ^*^P < 0.05, ^****^P < 0.0001 vs. C-exo mice, ^#^*P* < 0.05, ^###^*P* < 0.001 vs. T-exo mice (*n* = 6). **H** IF staining showed synaptopodin expression in mice. Scale bars: 10 μm. **I**–**K** IHC staining showed WT1 and podocin expression in mice. Scale bars: 25 μm. ^**^P < 0.01 vs. C-exo mice, ^#^*P* < 0.05 vs. T-exo mice (*n* = 6). Bonferroni’s correction was performed to analyze statistical significance. Data are the mean ± SEM.

As shown in Fig. 8A, B, miR-486-5p signals were mainly localized in the cytoplasm of glomerular cells and upregulated in DKD patients and db/db mice. Therefore, to further investigate the relevance of GEC-derived exosomes in podocyte injury and the crucial role of miR-486-5p in vivo, we carried out animal experiments using db/m mice by injecting TGF-β1-exo, TGF-β1+miR-486-inhibitor-exo, or Ctrl-exo isolated from HRGECs (Fig. 8C). As shown in Fig. 8D, PKH67-labeled exosomes were significantly delivered to glomerular podocytes after tail vein injection (Supplementary Fig. S7A). miR-486-5p was upregulated in mouse kidneys after injection with T-exo, which was reversed after injecting T+miR-in-exo (Fig. 8E). Additionally, pathological changes and renal functions were alleviated in T+miR-in-exo mice (Fig. 8F, G and Supplementary Fig. S7B, C). Most importantly, synaptopodin, WT1, podocin and PTEN expression was decreased in T-exo mice, which was reversed in T+miR-in-exo mice (Fig. 8H–K and Supplementary Fig. S7D). Conversely, the protein expression of p-AKT (S473) was elevated in T-exo mice and decreased in T+miR-in-exo mice (Supplementary Fig. S7E). Taken together, these results showed that GEC-derived exosomes induced by TGF-β1 mediated podocyte injury via miR-486-5p.

### Specific knockdown of miR-486-5p in GEC protects against podocyte injury in mice with diabetes mellitus

**Fig. 9:**
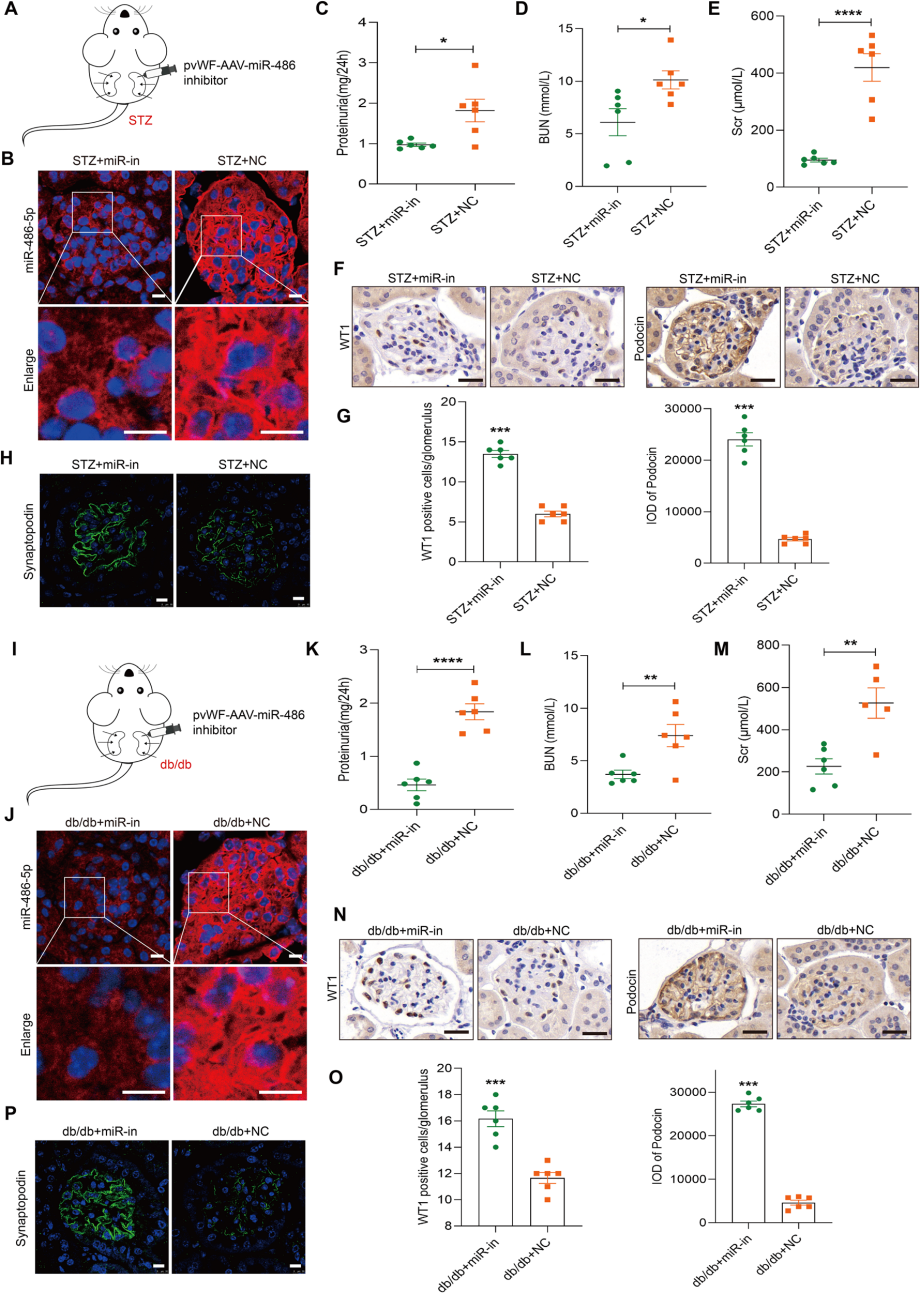
Specific knockdown of miR-486-5p in GECs protects against podocyte injury in vivo. **A** Twelve 20-week-old STZ-induced diabetic mice were randomly divided into two groups: STZ+miR-inhibitor (*n* = 6) and STZ + NC (*n* = 6). The mice were renally injected with 50 μl of 1 × 10^11^ infective units of adeno-associated virus at three sites in both kidneys. Four weeks later, the mice were sacrificed. **B** FISH assay showed miR-486-5p expression and localization in STZ mice. Scale bars: 10 μm. **C**–**E** Level of 24-h proteinuria, BUN, and Scr in mice. ^*^P < 0.05, ^****^P < 0.0001 vs. STZ + NC mice (*n* = 6). **F**, **G** IHC staining showed WT1 and podocin expression increased in STZ+miR-inhibitor mice. Scale bars: 25 μm. ^***^P < 0.001 vs. STZ + NC mice (*n* = 6). **H** IFstaining showed synaptopodin expression increased in STZ+miR-inhibitor mice. Scale bars: 10 μm. **I** Twelve 20-week-old db/db diabetes mice were randomly divided into two groups: db/db+miR-inhibitor (*n* = 6) and db/db+NC (*n* = 6). The mice were renally injected with 50 μl of 1 × 10^11^ infective units of adeno-associated virus at three sites in both kidneys. The mice were sacrificed after 4 weeks. **J** FISH assay showed miR-486-5p expression and localization in db/db mice. Scale bars: 10 μm. **K**–**M** Levels of 24-h proteinuria, BUN, and Scr in mice. ^**^P < 0.01, ^****^P < 0.0001 vs. db/db+NC mice (*n* = 6). **N**, **O** IHC staining showed WT1 and podocin expression increased in db/db+miR-inhibitor mice. Scale bars: 25 μm. ^***^P < 0.001 vs. STZ + NC mice (*n* = 6). **P** IF staining showed synaptopodin expression increased in db/db+miR-inhibitor mice. Scale bars: 10 μm. Student’s *t* test was performed to analyze statistical significance. Data are the mean ± SEM.

To further explore whether GEC-derived exosomes transferred miR-486-5p and mediated podocyte injury in vivo, STZ mice were renally injected with pvWF-AAV-miR-486-inhibitor (Fig. 9A). We found that specific knockdown of miR-486-5p expression in GECs relieved proteinuria, BUN, and Scr in STZ mice (Fig. 9B–E), which was accompanied by improved pathological changes (Supplementary Fig. S8A). Interestingly, inhibition of miR-486-5p upregulated synaptopodin, WT1, podocin and PTEN expression and downregulated p-AKT (S473) expression in STZ mice (Fig. 9F–H and Supplementary Fig. S8B, C). Similar results were observed in db/db mice (Fig. 9I–P).

## Discussion

Both GECs and podocytes are important components of the filtration membrane and their individual or simultaneous damage is closely related to proteinuria. However, the mechanism by which the initial injury of GECs drives podocyte injury remains unclear. In this study, we demonstrated a novel mechanism through which the exosomal miR-486-5p/PTEN axis mediated cellular communication between GECs and podocytes and subsequently induced proteinuria as well as elucidated a regulatory role for TRIM27 in crosstalk between GECs and podocytes. Our findings may provide a novel molecular target for the treatment of proteinuria.

First, our study confirmed TRIM27 overexpression in glomerular endothelial cells of both patients and mice with DKD and upregulation of TRIM27 mediated GEC injury by activating the JAK/STAT3 signaling pathway in DKD. Previous studies mainly focused on the role of TRIM27 in tumorigenesis and cancer progression, innate immunity, and viral replication [13, 14, 25–27]. More importantly, our previous study revealed that TRIM27 contributes to GEC injury and the proliferation of mesangial cell in glomeruli of lupus nephritis and in turn cause proteinuria [16, 28]. However, the exact role of TRIM27 in EC injury has not been investigated in DKD. This study clarified the role of TRIM27 in EC injury in DKD.

The most important finding of the current study was that podocyte injury was relieved in diabetic mice by inhibiting TRIM27-induced endothelial cell dysfunction. The interaction between GECs and podocytes has been considered to be closely related to the pathogenesis of DKD, especially the occurrence of proteinuria. However, the detailed molecular mechanism has not been fully illustrated. Lv et al. revealed the potential role of exosomes in kidney injury [29]. Our data also showed that HG or TGF-β1-treated HRGECs secreted more exosomes than those in the control group. Importantly, exosomes derived from injured GECs induced by HG or TGF-β1 promoted podocyte injury. Furthermore, pharmacological blockade of exosome biogenesis and secretion relieved podocyte injury in two models of DKD. The current study extended the prevalent assumption that soluble factors mediate podocyte injury in a paracrine manner [30–32], by providing evidence for the role of exosomes as a vehicle transporting genetic information and signals in mediating podocyte injury and proteinuria. Additionally, downregulating TRIM27 in GECs decreased exosomes secretion via reducing Rab27a expression. However, the mechanism of TRIM27 regulating Rab27a needs further investigation. The above findings provide novel insights into the pathomechanism of how cell–cell communication operates in the injured kidney. Therefore, exosomes can be used as a new target to prevent and treat DKD.

Exosomes are small vesicles that are released into the extracellular space and shuttle their cargo to neighboring or remote cells, thereby altering the behaviors of recipient cells [33]. Microarray analysis in our study revealed that miRNAs, especially miR-486-5p, were selectively loaded into exosomes in GECs treated with both HG and TGF-β1. Exosomes purified from cultured HRGECs in vivo had remarkably high expression of miR-486-5p compared with controls and induced podocyte injury. MicroRNAs are involved in podocyte injury by inhibiting protein synthesis or initiating mRNA degradation [34–36]. Additionally, miRNAs can be selectively enriched in secreted exosomes, transmit information, and affect the structure and function of distant target cells [36–38]. A previous study has suggested that exosomes can be incorporated by target cells [39]. In our study, PKH67-labeled HRGECs exosomes were internalized by HPCs. Therefore, miR-486-5p was delivered from HG- or TGF-β1-treated GECs to neighboring podocytes by exosomes and elevated miR-486-5p expression in podocytes, and inhibition of miR-486-5p in exosomes obviously attenuated podocyte injury, which supports the functional importance of miR-486-5p is in intercellular communication between GECs and podocytes. Recent studies found that miR-486-5p regulates the Akt signaling pathway and is involved in ischemia-reperfusion renal injury and lung cancer by targeting PTEN expression [23, 24, 40]. Moreover, the PTEN/Akt signaling pathway mediates a number of biological processes, including apoptosis, proliferation, differentiation, metabolism. We previously found that HG mediates podocyte apoptosis by activating the Akt signaling pathway in the early stage of diabetes [41]. In this study, we confirmed that miR-486-5p-containing exosomes mediated the podocyte injury via the PTEN/Akt axis. It is noteworthy that endothelial cell-derived exosomes mediated kidney injury of db/m mice in vivo, especially podocyte injury, whereas TGF-β1-exosomes devoid of miR-486-5p did not provoke podocyte injury. These data underlined an essential role of exosomes and miR-486-5p in the pathogenesis of DKD. More important, it was demonstrated in the current study that inhibition of miR-486-5p in endothelial cells significantly improved renal functions and decreased kidney injury in mice with STZ-induced diabetes. Furthermore, several studies found that exosomes regulate kidney diseases by transferring specific cytokines, mRNAs or miRNAs [42–44]. In this regard, we cannot rule out the possibility that other components in exosomes may also play a role in mediating kidney injury.

In summary, exosome mediated cell–cell communication plays an important role in regulating podocyte injury and proteinuria in DKD. TRIM27-mediated GEC injury triggers increased production of miR-486-5p-containing exosomes in glomerular endothelial cells. Delivery of endothelial cell-derived exosomes promotes podocyte injury and proteinuria. These results offer novel mechanistic insights into how cell–cell communication operates in DKD and provide a proof-of-principle that targeting the biogenesis and secretion of exosomes may be a new approach for therapeutics of DKD.

## Materials and methods

### Patients and samples

Thirty-six patients diagnosed with DKD were recruited from the inpatient at the Department of Nephrology, the Second Hospital of Hebei Medical University from 2019 to 2021, including 19 males and 17 females aged 30–55 years old. Percutaneous puncture was performed under ultrasound guidance for renal biopsy. Twenty-four normal renal tissues were obtained from patients with renal tumors during operations, such as renal clear cell carcinoma and renal leiomyoma, without a history of primary glomerulonephritis, hypertension, diabetic nephropathy, or other autoimmune diseases, and pathologically diagnosed with normal kidney tissue, and ages and genders consistent with the DKD group (Supplementary Fig. S1A). Renal tissues were fixed with 4% formaldehyde for hematoxylin and eosin (HE) staining, periodic acid-Schiff (PAS) staining, immunohistochemistry (IHC), and immunofluorescence (IF). Serum and urine samples were obtained for enzyme-linked immunosorbent assay (ELISA) and exosome isolation. The study was approved by the Clinical Research Ethics Committee of the Second Hospital of Hebei Medical University (IRB number: 20190040). Written informed consent was obtained from each study participant.

### Immunofluorescence

IF staining was carried out as described in our precious study [16]. The antibodies were anti-TRIM27 (1:200; Proteintech, Rosemont, IL, USA, #12205-1-AP, RRID: AB_2256660), anti-CD31 (1:100; Proteintech, #66065-2-Ig, RRID: AB_2918476), anti-syndecan-1 (SDC-1; 1:200; Abcam, Cambridge, MA, USA, #ab128936, RRID: AB_11150990), anti-VE-cadherin (1:200; CST, Danvers, MA, USA, #2500, RRID: AB_10839118), anti-ZO-1 (1:200; Proteintech, #21773-1-AP, RRID: AB_10733242), anti-synaptopodin (1:200; Proteintech, #21064-1-AP, RRID: AB_10733120), and anti-phosphatase and tensin homolog deleted on chromosome ten (PTEN; 1:200; Abcam, #ab32199, RRID: AB_777535). The images of sections and cells were captured under a laser scanning confocal microscope (Leica, Wetzlar, Germany). Image Pro Plus (Media Cybernetics, Silver Spring, MD) was used to quantify the results. The average integrated optical density (IOD) value was quantified to indicate protein expression.

### Enzyme-linked immunosorbent assay

The serum of DKD patients or mice and culture supernatants of primary human renal glomerular endothelial cells (HRGECs) were collected. The supernatant of HRGECs was centrifuged to remove floating cells and apoptotic bodies. The levels of syndecan-1 (ZCi Bio, Shanghai, China, #ZC-36203 and #ZC-37836) and vascular cell adhesion molecule-1 (VCAM-1; ZCi Bio, #ZC-35268 and #ZC-38858) were measured by ELISA kits in accordance with the instructions.

### Animal experiments

#### STZ-induced diabetic mice

Seven-week-old male CD1 mice were purchased from Beijing Charles River Laboratory Animal Technology Co. Ltd (Beijing, China). The mice weighting 20–25 g were fed in a standard environment with regular light/dark cycles and free access to water and food. After seven days of adaptation, 30 CD1 mice were induced to type 1 diabetes by a single intraperitoneal injection of streptozotocin (STZ; Sigma, St. Louis, USA, #18883-66-4) at 150 mg/kg [45, 46]. Individual mice with a blood glucose concentrations of 16.7 mM for three consecutive days were confirmed to be type 1 diabetec. Furthermore, six CD1 mice received an equal volume of vehicle (0.1 M citrate buffer, pH 4.5) as the control group. At 20 weeks, STZ-induced mice were randomly divided into five groups: STZ, STZ+pvWF-AAV-shNC (negative control, NC), STZ+pvWF-AAV-shTRIM27, STZ+pvWF-AAV-NC, and STZ+pvWF-AAV-miR486-inhibitor (pvWF is an endothelial cell specific promoter) groups. Mice in STZ+pvWF-AAV-shNC, STZ+pvWF-AAV-shTRIM27, STZ+pvWF-AAV-NC, and STZ+pvWF-AAV-miR486-inhibitor groups were renally injected with 50 μl 1 × 10^11^ infective units of adeno-associated virus (Hanbio Biotechnology Co., Ltd, Shanghai, China) at three sites each in both kidneys, whereas six control and six STZ mice were injected with isometric saline. Four weeks later, the mice were sacrificed after collecting the 24-h urine and blood samples, and the renal cortex was collected for relevant investigations. The 24-h proteinuria was detected by a mouse urine protein ELISA quantitation kit (ZCi Bio, #ZC-38527) in accordance with the manufacturer’s protocol. Blood samples were collected for blood glucose, blood urea nitrogen (BUN; Nanjing Jiancheng Bioengineering Institute, Nanjing, Jiangsu, China, #C013-2-1), serum creatinine (Scr; Nanjing Jiancheng Bioengineering Institute, #C011-2-1), SDC-1, and VCAM-1 measurements.

#### C57BLKS/J db/db mice

Seven-week-old male C57BLKS/J db/db mice (32–36 g) with genetic type 2 diabetes and db/m mice (26–28 g) were purchased from Cavens Biogle Model Animal Research Co. Ltd (Suzhou, Jiangsu, China). The db/m mice served as a control group, and 30 db/db mice aged 20 weeks old were randomly divided into five groups: db/db, db/db+pvWF-AAV-shNC, db/db+pvWF-AAV-shTRIM27, db/db+pvWF-AAV-NC, and db/db+pvWF-AAV-miR486-inhibitor groups. The following treatments was carried out in accordance with type 1 diabetic mice.

#### Exosome injection into C57BLKS/J db/m mice

Exogenous exosomes collected from control or transforming growth factor-β1 (TGF-β1)-treated HRGECs were injected intravenously into 10-week-old male db/m mice. To study the effects of miR-486-5p capsulated in the exosomes, the TGF-β1+miR-486-5p-inhibitor exosomes were also injected into db/m mice. After quantification by a micro bicinchoninic acid protein assay (Solarbio Life Sciences, Beijing, China, #PC0020), the same amounts of Ctrl-exo, TGF-β1-exo, and TGF-β1+miR-486-5p-inhibitor-exo (1 mg per mouse per time point) were respectively injected into mice via the tail vein at 1, 3, 5, 7, 9, 11, and 13 days.

All animals experiments were approved by the Institutional Animal Care and Use Committee of Hebei Medical University (approval ID: HebMU 2021043).

### Cell culture and treatment

Primary HRGECs were purchased from ScienCell (Carlsbad, CA, USA, #4000). Immortalized human glomerular podocytes (HPCs) were purchased from BeNa Culture Collection (Beijing, China). Cells were cultured at 37 °C in an incubator (Thermo Fisher Scientific, Waltham, MA, USA) with 5% CO_2_. HRGECs were cultured in endothelial cell medium (ScienCell, #1001) supplemented with 5% exosome-free fetal bovine serum (ScienCell, #1001), 1% penicillin/streptomycin solution (ScienCell, #1001), and 1% endothelial cell growth supplements (ScienCell, #1001), and used in the experiments at three to five passages. HPCs were cultured in RPMI 1640 medium (Thermo Fisher Scientific, #C11875500BT) supplemented with 10% exosome-free fetal bovine serum (Thermo Fisher Scientific, #10099) and 1% penicillin/streptomycin solution (Solarbio Life Sciences, #P1400).

1. To investigate the effect of high glucose (HG) and TGF-β1 on TRIM27 expression and injury of HRGECs, the HRGECs were randomly exposed to HG (30 mM) or TGF-β1 (20 ng/ml; MCE, New Jersey, USA, #HY-P7118) and collected at various times.
2. To further explore the role of TRIM27 in HRGEC injury, the HRGECs were randomly divided into eight groups: control, HG, mannitol (MA), HG+shTRIM27, HG+shNC, TGF-β1, TGF-β1+shTRIM27, and TGF-β1+shNC groups, and the cells were collected at 24 h after treatment with HG or TGF-β1.
3. To explore the significance of the JAK signaling pathway in TRIM27-induced HRGEC injury, the HRGECs were randomly divided into twelve groups: control, HG, MA, HG + AG490, HG + DMSO, HG+siSTAT3, HG+siNC, TGF-β1, TGF-β1 + AG490, TGF-β1 + DMSO, TGF-β1+siSTAT3, and TGF-β1+siNC groups. The cells were pretreated with AG490 (10 μM; MCE, #HY-12000) for 30 min and then stimulated with HG or TGF-β1. After 24 h, the cells were collected and related indicators were detected.
4. To further investigate crosstalk between the HRGECs and HPCs and the role of exosomes in this relationship, the HPCs were randomly divided into control, control conditioned medium (CM), HG conditioned medium (HM), MA conditioned medium (MM), TGF-β1 conditioned medium (TM), TGF-β1 + GW4869 conditioned medium (TM + GW4869), HG+shTRIM27 conditioned medium (HM+shTRIM27), HG+shNC conditioned medium (HM+shNC), TGF-β1+shTRIM27 conditioned medium (TM+shTRIM27), TGF-β1+shNC conditioned medium (TM+shNC), control exosome (C-exo), HG exosome (H-exo), MA exosome (M-exo), TGF-β1 exo (T-exo), HG+miR-486-5p inhibitor exosome (H+miR-in-exo), HG + NC exosome (H + NC-exo), TGF-β1+miR-486-5p inhibitor exosome (T+miR-in-exo), and TGF-β1 + NC exosome (T + NC-exo) groups. In accordance with previously described methods [19], after HRGECs were treated with HG or TGF-β1 for 24 h, stimuli were removed, followed by incubation in control medium for 24 h. In some experiments, HRGECs were pretreated with GW4869 (10 μM; APExBIO, Houston, Texas, USA, #C4769) for 2 h to explore the role of exosomes or transfected with TRIM27 shRNA and a miR-486-5p inhibitor to explore the role of TRIM27 and miR-486-5p. Conditioned media were collected and subjected to exosome isolation. Then, the HPCs were treated with HRGEC-conditioned media or exosomes (30 μg protein/ml) for 48 h, and various subsequent analyses were performed as described.
5. To investigate whether the PTEN/Akt pathway participated in HPC injury, Akt inhibitor LY294002 was used. The HPCs were pretreated with LY294002 (10 μM; APExBIO, #A8250) for 60 min or overexpressed PTEN and then stimulated by exosomes isolated from HRGECs treated with TGF-β1 or HG for 48 h.

### NO measurement

HRGECs were lysed at 4 °C for 1 h to generate the nitric oxide lysate and then centrifuged at 12,000 *g* for 20 min. The supernatants were collected to measure the NO contents using a Nitric Oxide Detection Kit (Beyotime, Shanghai, China, #S0021S) in accordance with the manufacturer’s instructions.

### Plasmids and transfection

HRGECs were transfected with TRIM27-shRNA (Cyagen Biotechnology Co., Ltd, Santa Clara, California, USA), WT-PTEN (Cyagen Biotechnology Co., Ltd) or miR-486-5p inhibitor (RIBOBIO, Guangzhou, China, #miR20002177) using lipofectamine 3000 (Invitrogen, Carlsbad, CA, USA, #L3000015) according to the manufacturer’s protocols.

### Western blotting

Total protein from HRGECs and HPCs was extracted in RIPA lysis buffer. Equal amounts of proteins (30 μg) were separated by 10% or 12% SDS-PAGE and then transferred to PVDF membranes (Millipore, Billerica, MA, USA). The membranes were blocked with 5% BSA for 1.5 h at 37 °C. Then, the membranes were incubated overnight at 4 °C with 1:1000 dilutions of antibodies against TRIM27 (Proteintech, #12205-1-AP), VCAM-1 (Abcam, #ab134047, RRID: AB_2721053), Janus kinase 2 (JAK2; Proteintech, #17670-1-AP, RRID: AB_2811138), p-JAK2 (Y1007) (Abcam, #ab195055), signal transducer and activator of transcription 3 (STAT3; Proteintech, #60199-1-Ig, RRID: AB_10913811), p-STAT3 (Tyr705) (CST, #9145, RRID: AB_2491009), nephrin (Abcam, #ab58968, RRID: AB_944400), podocin (Abcam, #ab50339, RRID: AB_882097), CD63 (Santa Cruz Biotechnology, Dallas, TX, USA, #sc-5275, RRID: AB_627877), CD9 (Santa Cruz Biotechnology, #sc-13118, RRID: AB_627213), TSG101 (Proteintech, #28283-1-AP, RRID: AB_2881104), Calnexin (Proteintech, #10427-2-AP, RRID: AB_2069033), Rab27a (Proteintech, #17817-1-AP, RRID: AB_2176728), PTEN (Abcam, #ab32199, RRID: AB_777535), Akt (Proteintech, #60203-2-Ig, RRID: AB_10912803), and p-Akt (S473) (Abcam, #ab81283, RRID: AB_2224551). The next day, the membranes were incubated with horseradish peroxidase-conjugated goat anti-rabbit or mouse IgGs (1:5000; Proteintech, #SA00001-2, RRID: AB_2722564 and #SA00001-1, RRID: AB_2722565). After washing, the images were captured using the LI-COR Odyssey Infrared Imaging System (Lincoln, NE, USA). All experiments were repeated at least three times.

### PAS staining

After dewaxing and rehydration, 2-μm-thick paraffin-embedded sections were stained with PAS. Light microscopy (OLYMPUS, Tokyo, Japan, BX71) was used to observe morphological changes in the glomeruli, including the number of cells, thickness of the basement membrane, and extracellular matrix deposition in mesangial areas.

### TMT-labeled quantitative proteomic analysis

The TMT-labeled quantitative proteomic analysis encompassed the extraction of total protein from samples, determination of protein concentration, Filter Aided Proteome Preparation (FASP) for trypsin digestion of each sample and subsequent labeling, chromatographic separation, as well as LC-MS/MS analysis and data interpretation, all conducted by APPLIED PROTEIN TECHNOLOGY Co., Ltd. (Shanghai, China).

### Immunohistochemistry

Renal tissue sections fixed in 4% formaldehyde were deparaffinized in xylene and rehydrated through graded ethanol solutions. After antigen retrieval in an autoclave, endogenous peroxidase was blocked with 3% H_2_O_2_ for 30 min at room temperature. Then, the sections were blocked with 10% goat serum and incubated with primary antibodies against Wilms tumor 1 (WT1; 1:200; Abcam, #ab89901, RRID: AB_2043201), p-Akt (S473) (1:200; Abcam, #ab81283) or podocin (1:200; Abcam, #ab50339) overnight at 4 °C. Then the sections were incubated with polymer helper and polyperoxidase-anti-mouse/rabbit IgG at 37 °C, and finally stained with diaminobenzidine. Images were captured under a BX71 microscope (OLYMPUS). The average IOD value was quantified to indicate protein expression by Image Pro Plus (Media Cybernetics).

### Electron microscopy

The renal cortex of mice cut into 1 mm^3^ cubes and exosomes isolated from HRGECs were fixed in 2.5% glutaraldehyde. The ultrastructures of podocytes in the renal cortex and cup-shaped membrane vesicles of the exosome was observed by transmission electron microscopy (TEM; HITACHI, Tokyo, Japan, HI7800).

### Exosome extraction

Exosomes were isolated from conditioned media using an Exosome Extraction Kit (BestBio, Shanghai, China, #BB-3901) in accordance with the manufacturer’s instructions. Briefly, conditioned media were centrifuged at 3000 *g* for 15 min, followed by 10,000 *g* for 20 min, and then the supernatant was incubated overnight with Extraction Buffer A. After centrifugation at 10,000 *g* for 60 min, the exosome pellets were resuspended in phosphate-buffered saline (PBS). All steps were performed at 4 °C. Finally, the exosomes were used for identification and quantification. Intact cup-shaped membrane vesicles were visualized by TEM, with their sizes matching the measurements from nanoparticle tracking analysis. Additionally, some exosomes were used to stimulate HPCs and injected into the tail vein of mice.

### Nanoparticle tracking analysis

The number and size of exosomes were directly tracked using an NS300 instrument (Malvern Instruments Ltd., Worcestershire, UK) equipped with a 633 nm laser and high-sensitivity sCMOS camera. The settings were a particle size range of 50–200 nm, molecular weight range of 1000–20107 Da, and temperature of 25 °C. Each sample was analyzed at least three consecutive times. Data were analyzed using NanoSight NTA data analysis software (Brookhaven Instruments Corporation, New York, USA).

### Fluorescent labeling of exosome

Exosomes were labeled with lipophilic dyes PKH67 (BestBio, #BB-3986) for 30 min and then washed three times with PBS. The PKH67-labeled exosomes were incubated with HPCs for 48 h or injected into mice via the tail vein and detected by IF.

### RNA extraction and qPCR

Total RNA was extracted using TRIzol RNA isolation reagent (TIANGEN, Beijing, China, #DP424). Reverse transcription was performed using PrimeScript^TM^ RT Master Mix (Takara, Japan, #047 A). Quantitative polymerase chain reaction (qPCR) was performed with SYBR Premix EX Taq (Takara, #820 A) in accordance with the manufacturer’s protocol. The 2^−ΔΔCT^ method was used to normalize the qPCR cDNAs. All experiments were repeated at least in triplicate.

### Dual luciferase reporter assay

HEK293T cells and HPCs were seeded in 24-well plates at 6 × 10^4^ cells per well at 24 h before transfection. Then, the cells were cotransfected with a mixture of miR-486-5p minics (20 nM, RIBOBIO, #miR10002177) and luciferase reporter vectors (psiCHECK^TM^-2 Vector) containing PTEN-binding sequences or mutant sequences (Fig. 7k). After 48 h, luciferase activity was measured using a Dual-Luciferase Reporter Assay System (Promega, Madison, WI, USA, #E1910) according to the manufacturer’s protocols. Luciferase activity was averaged from five replicates per transfection with three replicates of transfection.

### RNA fluorescence in situ hybridization (FISH)

Renal tissue sections fixed in 4% formaldehyde were deparaffinized in xylene and rehydrated through graded ethanol solutions. A specific probe for miR-486-5p was designed and synthesized (Focobio, Guangdong, Guangzhou, China) and its signals were detected by a FISH Kit (Focobio, #D-2922B) in accordance with the manufacturer’s protocols. Images were captured under the laser scanning confocal microscope (Leica).

### Statistical analysis

Results are expressed as the mean ± standard error of mean (SEM). SPSS 21.0 (SPSS, Inc., Chicago, IL) was used for data analysis. Student’s *t* test was applied to compare variables between two groups. One-way analysis of variance was performed to evaluate the statistical significance between multiple comparisons by Bonferroni’s correction. Pearson’s method was used for correlation analysis. *P* < 0.05 was considered statistically significant.

## Supplementary information

Supplementary Material

Original Western blots

## Data Availability

All relevant data are available within the manuscript and the Supplementary Materials. Requests for materials should be addressed to Y.T. (doctoryuexin@163.com) or S.L. (shuxialiu@hebmu.edu.cn).
