# Supplementary material for: TRIM27-controlled endothelium-derived exosomes play a central role in podocyte injury in diabetic kidney disease
Source: Cell Death Discov. 2026 Mar 7;12:138. doi: 10.1038/s41420-026-02953-y (PMC13039385; doi:10.1038/s41420-026-02953-y)
Supplement: Supplementary file 1 — Supplementary Material [file 41420_2026_2953_MOESM1_ESM.pdf]

SUPPLEMENTARY MATERIALS

TRIM27-controlled endothelium-derived exosomes play a central role in podocyte injury in diabetic kidney disease

Yuexin Tian[1*], Yunhe Liu[1*], Xiaojuan Feng[1*], Lunbi Wu[1], Weiwei Song[1], Tongyu Zhao[1], Jinxi Liu[1], Xinyan Miao[1], Haimin Ma[1],Baiyun Jia[1], Lihua Kang[1], Qingjuan Liu[1], Wei Zhang[1], Huifang Guo[2], Lin Yang[3], Jinsheng Xu[4#], Shuxia Liu[1#]

*Equal contributors.

#CO-corresponding Authors

Prof. Shuxia Liu, Department of Pathology, Hebei Key Laboratory of Nephrology, Center of Metabolic Diseases and Cancer Research, Institute of Medical and Health Science, Hebei Medical University, Shijiazhuang 050017, China, Fax: +86-311-86266942, Tel: ±86 311 86266584, E-mail: shuxialiu@hebmu.edu.cn

Prof. Jinsheng Xu, Department of Nephrology, Hebei Key Laboratory of Vascular Calcification in Kidney Disease, Hebei Clinical Research Center for Chronic Kidney Disease, The Fourth Hospital of Hebei Medical University, Shijiazhuang 050011, China, Fax: +86-311-86266942, Tel: ±86 311 86095878, E-mail: xjs5766@126.com.

Supplementary Figure S1

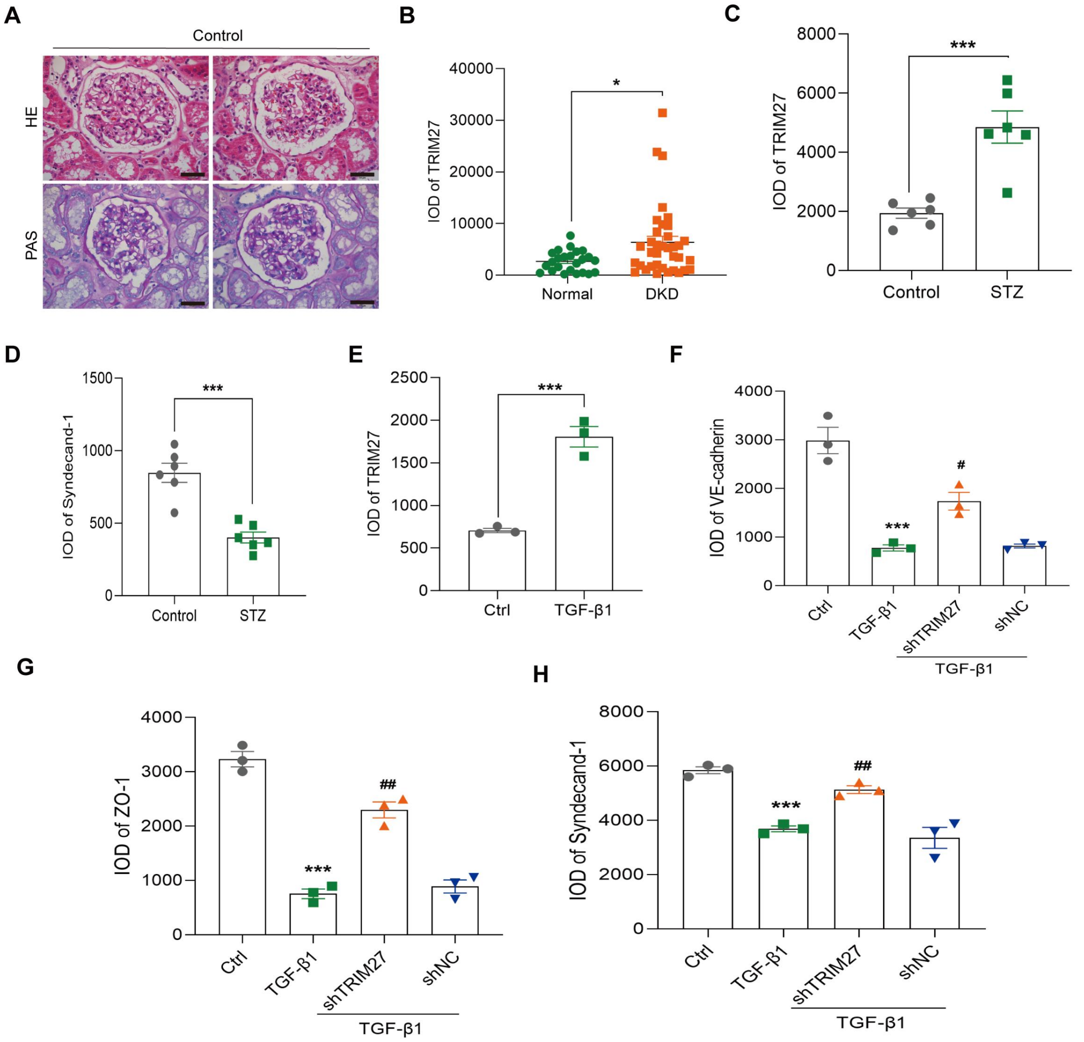

Supplemental Figure 1 (**A**) Distal noncancerous kidney tissues pathologically confirmed as normal tissues. Scale bars: 50 μm. (**B**) IOD of TRIM27 protein in glomerular cells of DKD patients. $^{*}P < 0.05$ vs. Normal group (n=24 normal controls and 36 DKD patients). (**C, D**) IOD of TRIM27 and syndecan-1 protein in glomerular cells of STZ mice. $^{***}P < 0.001$ vs. Control mice, n=6 each group. (**E-H**) IOD of TRIM27, VE-cadherin, ZO-1, and syndecan-1 protein in HRGECs treated with TGF-β1 for 24 h. $^{***}P < 0.001$ vs. control group, $^{\#}P < 0.05$, $^{\#\#}P < 0.01$ vs. TGF-β1+shNC group (n=3). Student's t-test and Bonferroni's correction were performed to analyze statistical significance. Values are the mean ± SEM.

Supplementary Figure S2

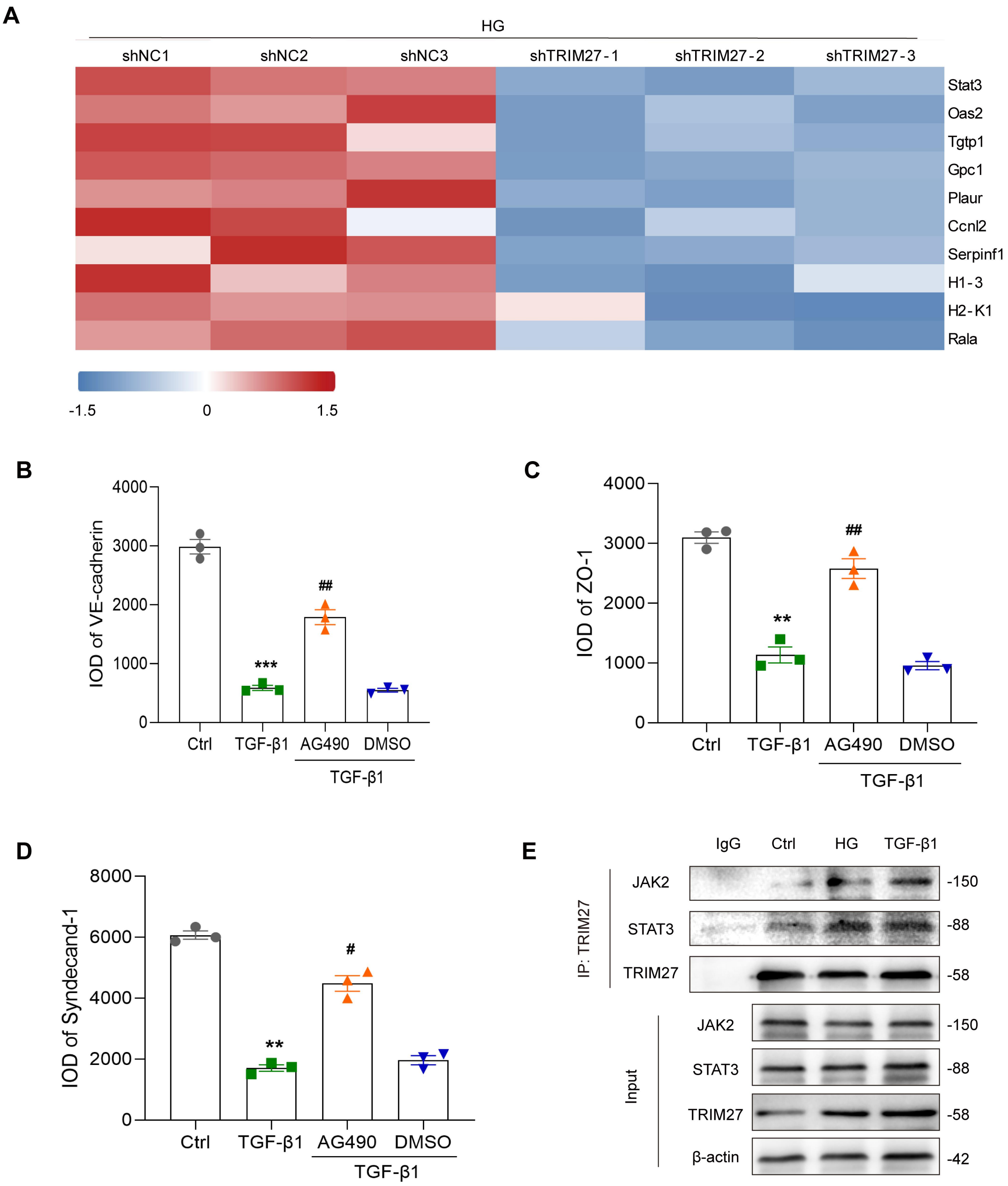

Supplemental Figure 2 TRIM27 regulates HRGEC injury caused by HG or TGF-β1 by activating the JAK2/STAT3 signal pathway. (**A**) Heatmap of 10 differentially expressed proteins between HG+shNC and HG+shTRIM27 HRGECs. (**B-D**) IOD of VE-cadherin, ZO-1, and syndecan-1 protein in HRGECs treated with TGF-β1. $^{**}P < 0.01$, $^{***}P < 0.001$ vs. control group, $^{\#}P < 0.05$, $^{\#\#}P < 0.01$ vs. TGF-β1+DMSO group (n=3). (**E**) Coimmunoprecipitation showed the interaction between TRIM27, JAK2 and STAT3 in HRGECs. Bonferroni's correction was performed to analyze statistical significance. Values are the mean ± SEM.

Supplementary Figure S3

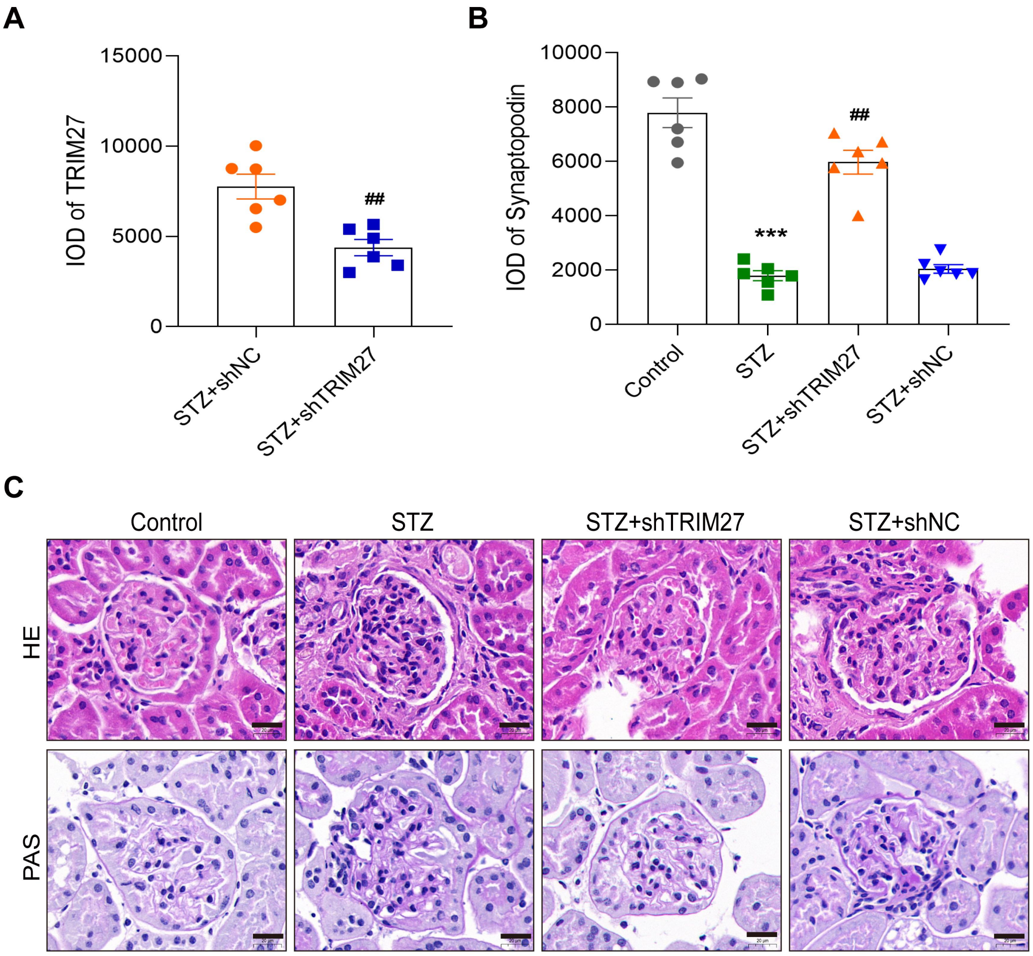

Supplemental Figure 3 (**A, B**) IOD of TRIM27 and syndecan-1 protein in glomerular cells of STZ mice. $^{***}P < 0.001$ vs. Control mice, $^{\#\#}P < 0.01$ vs. STZ+shNC mice, n=6 each group. (**C**)HE and PAS staining showed larger renal glomeruli volume, inflammatory cells infiltrate the interstitium, mesangial cell expansion, and matrix accumulation in STZ mice. However, pathological changes were alleviated by knockdown of TRIM27. Scale bars: 20 µm. Student's t-test and Bonferroni's correction were performed to analyze statistical significance. Values are the mean ± SEM.

Supplementary Figure S4

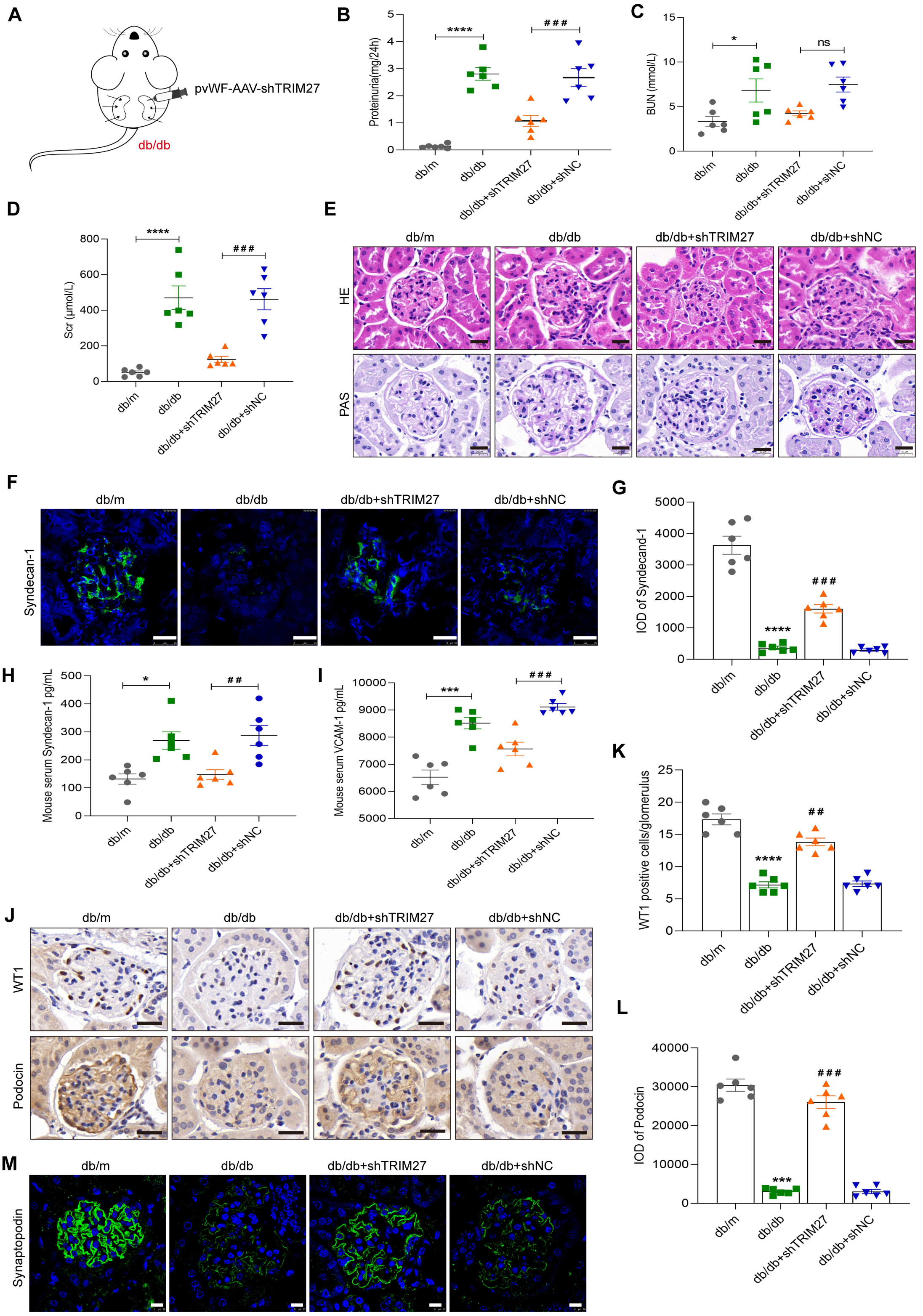

Supplemental Figure 4 Specific knockdown of TRIM27 expression in GECs suppresses GEC and podocyte injury in db/db mice. (**A**) Eighteen 20-week-old db/db mice were randomly divided into three groups: db/db (n=6), db/db+shTRIM27 (n=6),

and db/db+shNC (n=6). Mice in db/db+shTRIM27 and db/db+shNC group were renally injected with 50 µl of $1{\times}10^{11}$ infective units of adeno-associated virus at three sites in both kidneys. Six db/m mice and 6 db/db mice were injected with isometric saline. The mice were sacrificed after 4 weeks. (**B–D**) Levels of 24-h proteinuria, BUN, and Scr in mice. $^{*}P < 0.05$, $^{****}P < 0.0001$ vs. db/m mice, $^{###}P < 0.001$ vs. db/db+shNC mice, ns, no significance (n=6). (**E**) HE and PAS staining showed that specific knockdown of TRIM27 expression in GECs of db/db mice could suppressed larger renal glomeruli volume, inflammatory cells infiltrate the interstitium, mesangial cell expansion, and matrix accumulation in db/db mice. Scale bars: 20 µm. (**F, G**) IF staining showed syndecan-1 expression in mice. Scale bars: 25 µm. $^{****}P < 0.0001$ vs. db/m mice, $^{###}P < 0.001$ vs. db/db+shNC mice (n=6). (**H, I**) The ELISA assays showed the serum contents of syndecan-1 and VCAM-1 in of mice. $^{*}P < 0.05$, $^{***}P < 0.001$ vs. db/m mice, $^{##}P < 0.01$, $^{###}P < 0.001$ vs. db/db+shNC mice. n=6 each group. (**J–L**) IHC staining showed WT1 and podocin expression in mice. Scale bars: 25 µm. $^{***}P < 0.001$, $^{****}P < 0.0001$ vs. db/m mice, $^{##}P < 0.01$, $^{###}P < 0.001$ vs.db/db+shNC mice (n=6). (**M**) IF staining showed synaptopodin expression in mice. Scale bars: 10 µm. Bonferroni's correction was performed to analyze statistical significance. Values are the mean ± SEM.

Supplementary Figure S5

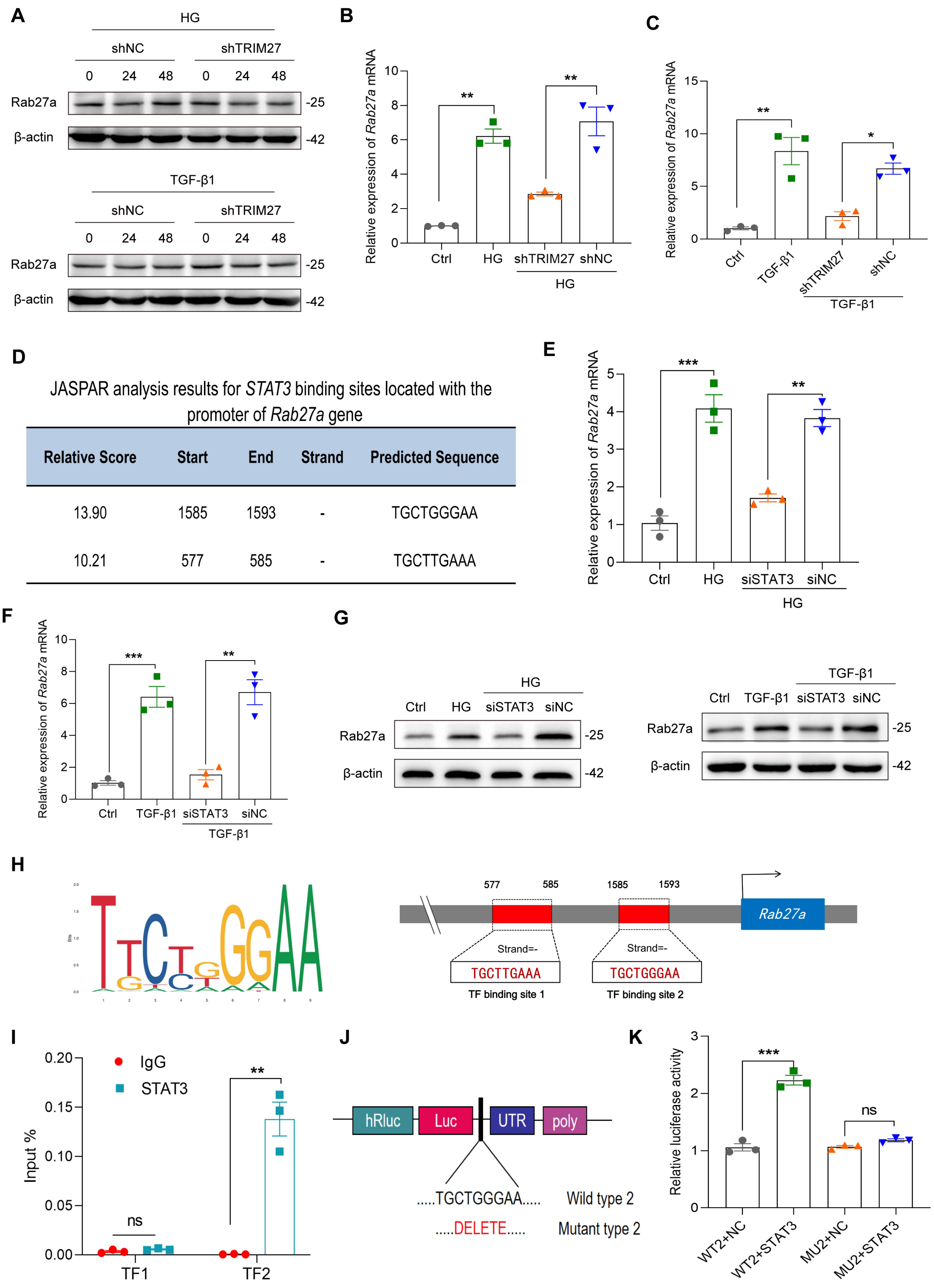

Supplemental Figure 5 TRIM27 regulates Rab27a by mediating the activation of its transcription factor STAT3. (**A**) Western blot assay showed Rab27a expression remained unchanged in TRIM27-knockdown HRGECs treated with 200 μg/mL CHX

at indicated time. (**B, C**) The qPCR assay showed the relative mRNA of Rab27a in HRGECs. (**D**) The JASPAR predicted and analyzed that STAT3 protein is a transcription factor of the Rab27a gene. (**E, F**) The qPCR assay showed the relative mRNA of Rab27a in HRGECs. **(G)** Western blot assay showed Rab27a expression decreased in STAT3-knockdown HRGECs treated with HG or TGF-β1 for 24 h. **(H)** JASPAR predicted a conserved STAT3-binding motif, with schematics showing the potential STAT3 binding sites in the Rab27a promoter. **(I)** ChIP analysis of STAT3 occupancy at the Rab27a promoter in HRGECs. **(J)** Schematic diagrams of luciferase reporter plasmids harboring either the wild-type (WT2) or mutant (MUT2) STAT3 binding sites within the Rab27a promoter region. **(K)** Dual luciferase reporter assay was performed to confirm the binding site of STAT3 and Rab27a in 293T cells. $^{*}P < 0.05$, $^{**}P < 0.01$, $^{***}P < 0.001$, and ns: no significant. Student's t-test and Bonferroni's correction were performed to analyze statistical significance. Values are the mean ± SEM.

Supplementary Figure S6

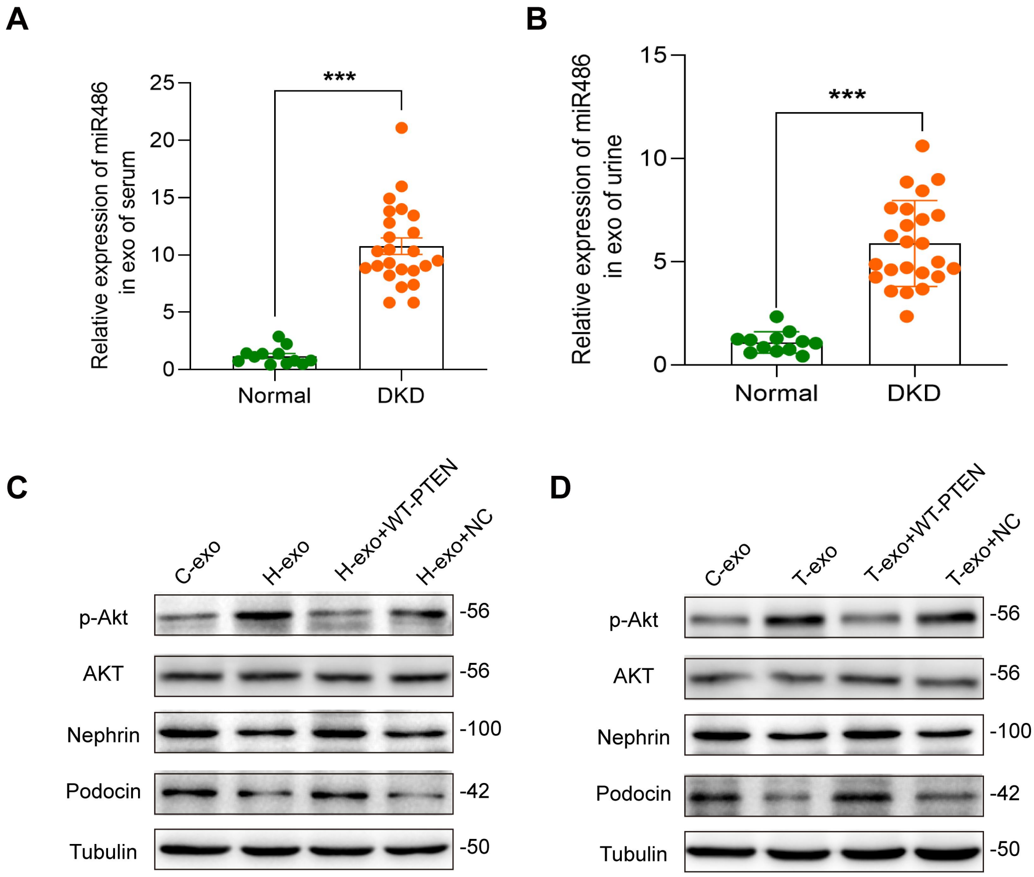

Supplemental Figure 6 (**A, B**) Detection of miR-486-5p in serum and urine of DKD patients by qPCR normalized to U6. $^{***}P < 0.001$ vs. Normal group (n=24 normal controls and 36 DKD patients). **(C, D)** Western blot assay showed p-AKT (S473), nephrin and podocin expression in the HPCs treated with exosomes. Student's t-test was performed to analyze statistical significance. Values are the mean ± SEM.

Supplementary Figure S7

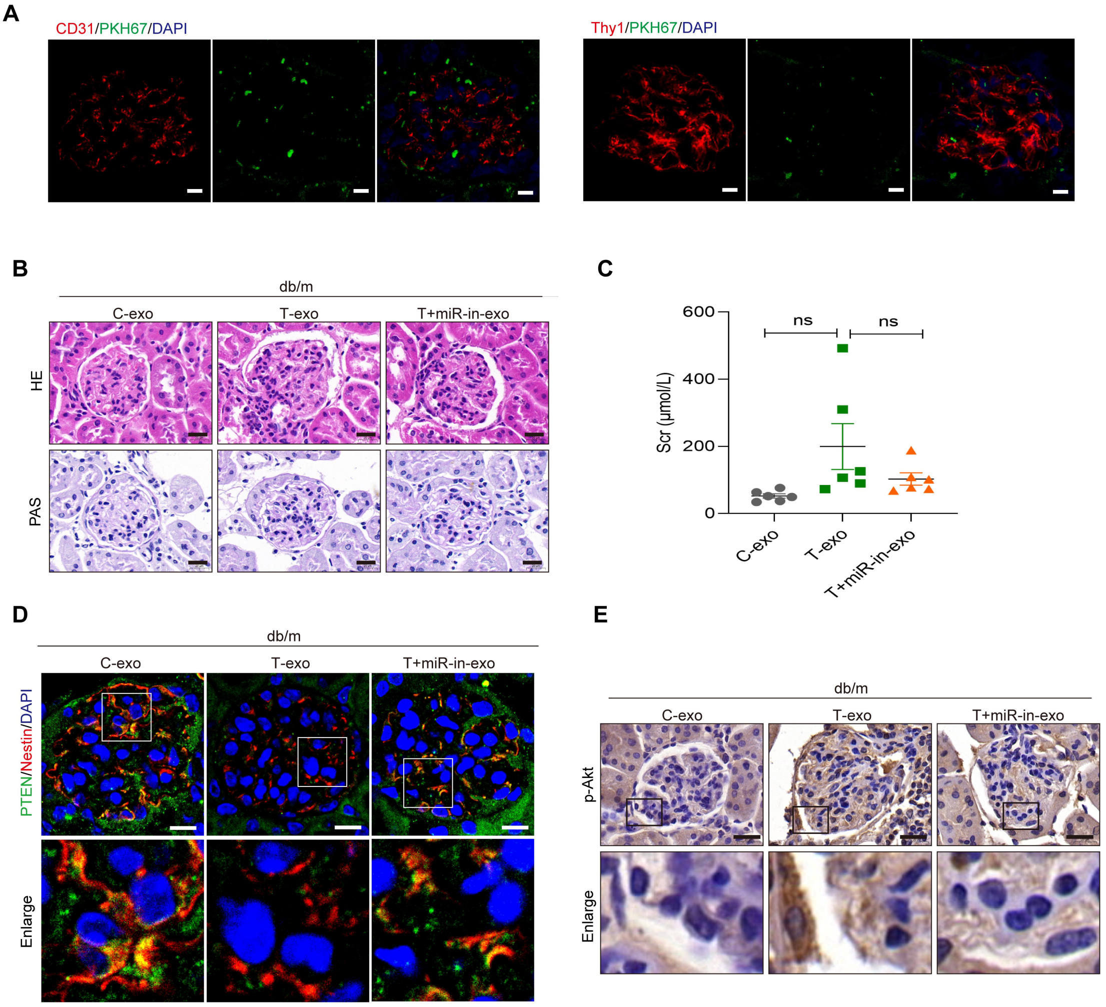

Supplemental Figure 7 (**A**) IF showed PKH67-labeled exosomes in mouse kidneys after i.v. injection. CD31 (green) was used as a endothelial cells-specific marker. Thy1 (green) was used as a mesangial cells-specific marker. Scale bars: 10 μm. **(B)** HE and PAS staining showed pathological changes were alleviated in T+miR-in-exo mice. Scale bars: 20 μm. **(C)** Level of Scr in mice. ns, no significance (n=6). **(D)** Double immunofluorescence staining of PTEN protein in podocytes of mice. Kidney sections were costained for PTEN (green) and specific podocyte marker nestin (red). Scale bars: 50 μm. **(E)** IHC staining showed p-AKT (S473) expression in mice. Scale bars: 20 μm. Bonferroni's correction was performed to analyze statistical significance. Values are the mean ± SEM.

Supplementary Figure S8

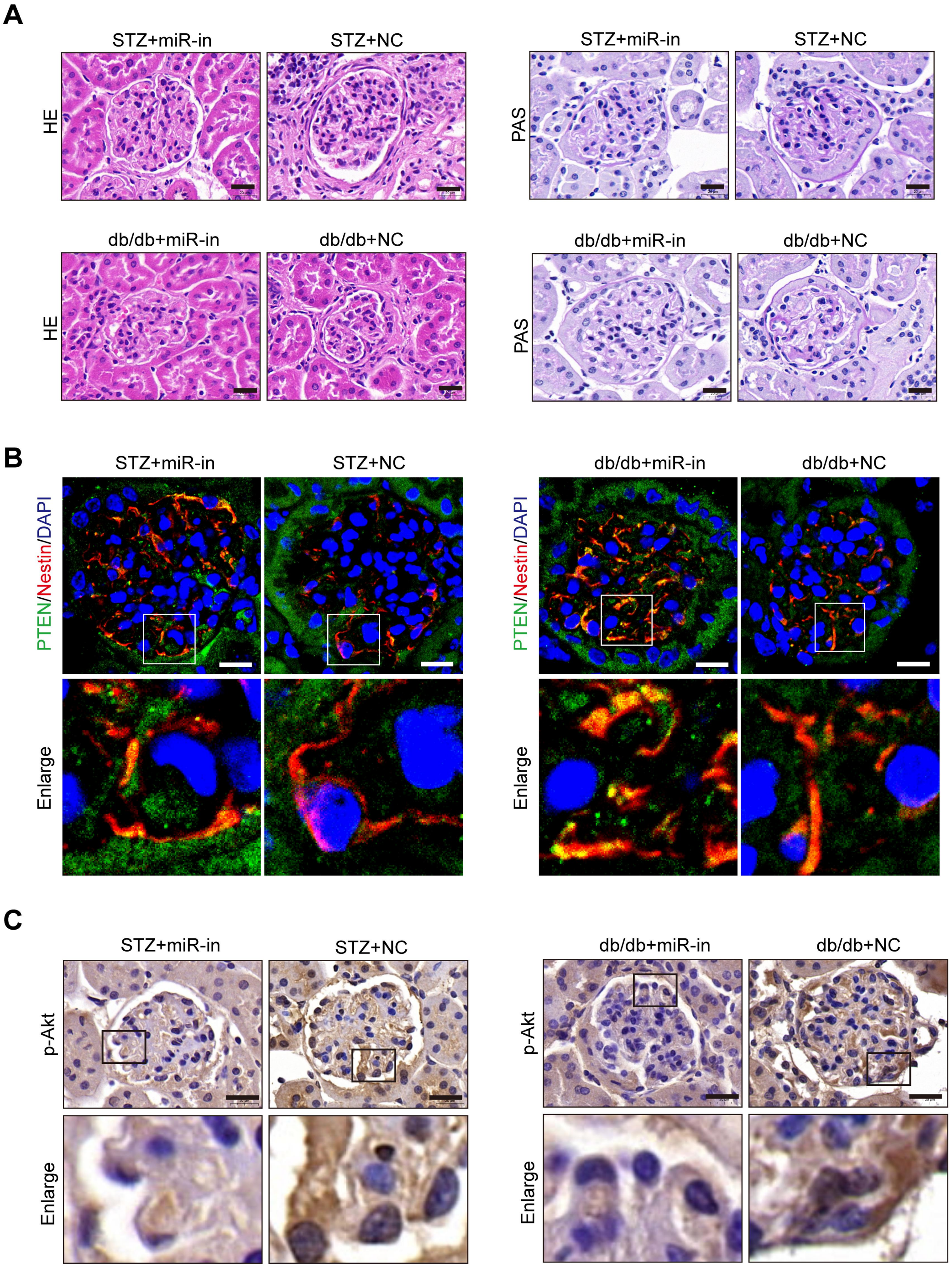

Supplemental Figure 8 (**A**) HE and PAS staining showed that specific knockdown of miR-486-5p expression in GECs suppressed the glomeruli injury in mice. Scale bars: 20 μm. **(B)** Double immunofluorescence staining of PTEN protein in podocytes of mice. Kidney sections were costained for PTEN (green) and specific podocyte marker nestin (red). Scale bars: 50 μm. **(C)** IHC staining showed p-AKT (S473) expression in mice. Scale bars: 20 μm.

Table S1 Physical and biochemical parameters of STZ-induced mice

Variables	Control	STZ	STZ+shTRIM27	STZ+shNC
BG (mmol/L)	5.87±0.21	22.95±0.24*	22.15±0.20*	22.98±0.19*
BW (g)	25.72±0.37	22.85±0.32*	23.33±0.18*	22.65±0.30*
KW/BW (mg/g)	8.97±0.13	15.37±0.20*	12.76±0.09*#	15.41±0.22*
Proteinuria (mg/24h)	0.15±0.04	1.63±0.21*	0.76±0.10#	1.85±0.27*
BUN (mmol/L)	3.02±0.24	8.99±0.75*	2.53±0.34#	7.72±0.58*
Scr (μmol/L)	72.91±12.76	387.90±39.98*	126.10±13.53#	370.00±15.68*

BG, blood glucose; BW, body weight; KW/BW, kidney weight/body weight; BUN, blood urea nitrogen; Scr, serum creatinine.
Data are expressed as means ± SEM. *$P < 0.05$ vs. Control mice, #$P < 0.05$ vs. STZ+shNC mice.

Table S2 Physical and biochemical parameters of db/db mice

Variables	db/m	db/db	db/db+shTRIM27	db/db+shNC
BG (mmol/L)	5.90±0.33	25.07±0.65*	24.48±0.51*	25.40±0.76*
BW (g)	25.67±0.26	52.73±0.99*	52.53±1.09*	52.45±1.24*
KW/BW (mg/g)	5.59±0.08	5.15±0.15	5.03±0.10	5.16±0.12
Proteinuria (mg/24h)	0.13±0.03	2.81±0.23*	1.08±0.20*#	2.67±0.33*
BUN (mmol/L)	3.34±0.55	6.82±1.30*	4.25±0.28	7.48±0.84*
Scr (μmol/L)	50.36±8.96	469.90±66.40*	123.20±16.97#	461.70±59.18*

BG, blood glucose; BW, body weight; KW/BW, kidney weight/body weight; BUN, blood urea nitrogen; Scr, serum creatinine.
Data are expressed as means ± SEM. *$P < 0.05$ vs. db/m mice, #$P < 0.05$ vs. db/db+shNC mice.

Table S3 Physical and biochemical parameters of HRGEC-derived exosomes-induced db/m mice

Variables	db/m+C-exo	db/m+T-exo	db/m+T-miR-in-exo
BG (mmol/L)	5.89±0.30	5.65±0.23	5.52±0.37
BW (g)	25.60±0.33	25.40±0.28	25.53±0.56
KW/BW (mg/g)	5.91±0.17	5.92±0.12	5.84±0.22
Proteinuria (mg/24h)	0.28±0.07	1.14±0.14*	0.50±0.05#
BUN (mmol/L)	0.62±0.22	4.83±1.80*	0.25±0.10#
Scr (μmol/L)	52.04±6.47	199.50±68.32	103.00±18.29

BG, blood glucose; BW, body weight; KW/BW, kidney weight/body weight; BUN, blood urea nitrogen; Scr, serum creatinine.

Data are expressed as means ± SEM. *$P < 0.05$ vs. db/m+C-exo mice, #$P < 0.05$ vs. db/m+T-exo mice.

Table S4 Physical and biochemical parameters of STZ-induced mice

Variables	BG (mmol/L)	BW (g)	KW/BW (mg/g)	Proteinuria (mg/24h)	BUN (mmol/L)	Scr (μmol/L)
STZ+miR-in	22.70±0.56	23.00±0.26	14.64±0.16*	0.98±0.04*	6.11±1.29*	95.34±6.65*
STZ+NC	22.90±0.37	22.58±0.26	15.54±0.28	1.82±0.28	10.13±0.86	419.90±48.26

BG, blood glucose; BW, body weight; KW/BW, kidney weight/body weight; BUN, blood urea nitrogen; Scr, serum creatinine.

Data are expressed as means ± SEM. *$P < 0.05$ vs. STZ+NC mice.

Table S5 Physical and biochemical parameters of db/db mice

Variables	BG (mmol/L)	BW (g)	KW/BW (mg/g)	Proteinuria (mg/24h)	BUN (mmol/L)	Scr (μmol/L)
db/db+miR-in	25.12±0.42	52.05±0.70	5.06±0.13	0.46±0.11*	3.70±0.40*	226.90±36.41*
db/db+NC	25.40±0.56	52.83±0.89	4.86±0.06	1.84±0.15	7.40±1.06	526.80±71.97

BG, blood glucose; BW, body weight; KW/BW, kidney weight/body weight; BUN, blood urea nitrogen; Scr, serum creatinine.

Data are expressed as means ± SEM. **P* < 0.05 vs. db/db+NC mice.

Table S6 Prime sequence of genes

Primers	Forward (5'-3')	Reverse (5'-3')
miR-486-5p	ACATGCAATTTCCTGTACTGAGC	TATGGTTGTTCTCGTCTCTGTGTC
U6	GGAACGATACAGAGAAGATTAGC	TGGAACGCTTCACGAATTTGCG
Rab27a	GCTTTGGGAGACTCTGGTGTA	TCAATGCCCACTGTTGTGATAAA
GAPDH	GATTCCACCCATGGCAAATTC	CTGGAAGATGGTGATGGGATT
Rab27a-TF1	GAGTTTGGTACTGTCTACAG	CAGATGAATACTGGCAGTCT
Rab27a-TF2	ACAGCCTGTCGGCCAAATAC	CCGTTGAGCTCTGTGTTGCC
