# Supplementary material for: TRIM27-controlled endothelium-derived exosomes play a central role in podocyte injury in diabetic kidney disease
Source: Cell Death Discov. 2026 Mar 7;12:138. doi: 10.1038/s41420-026-02953-y (PMC13039385; doi:10.1038/s41420-026-02953-y)

Figure 1j

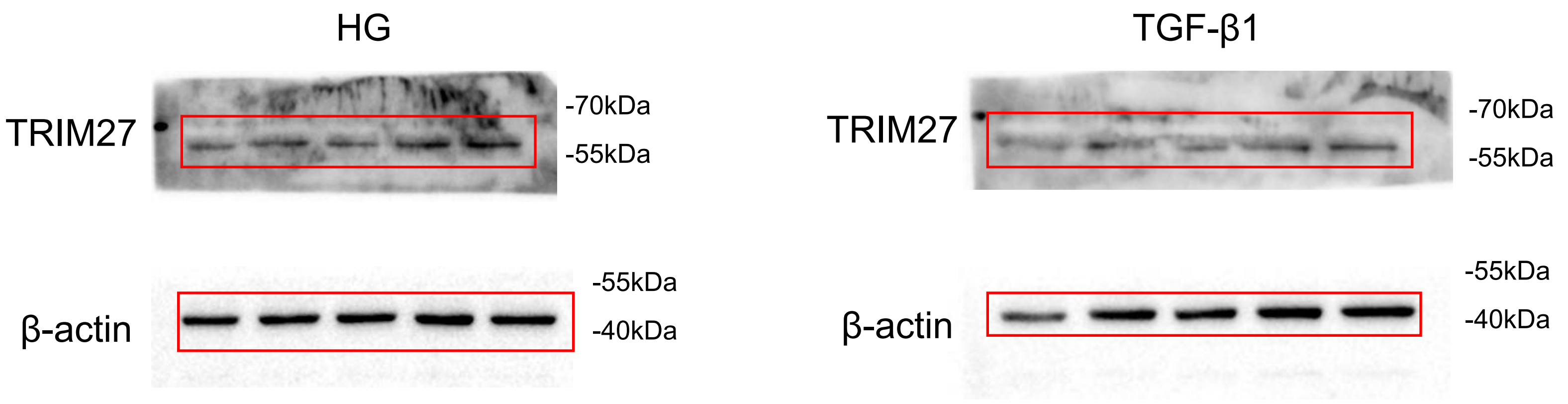

Figure 1n

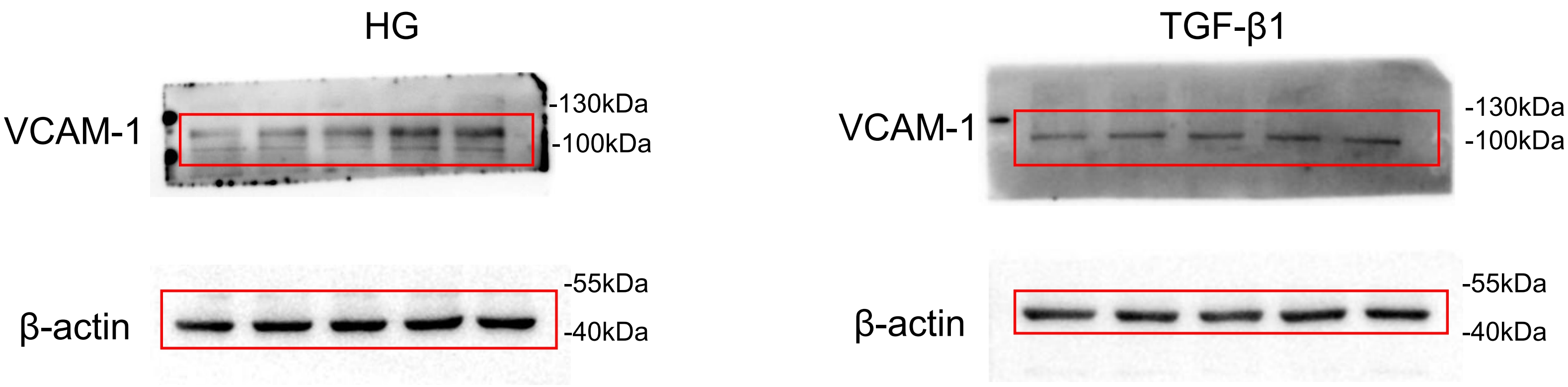

Figure 2a

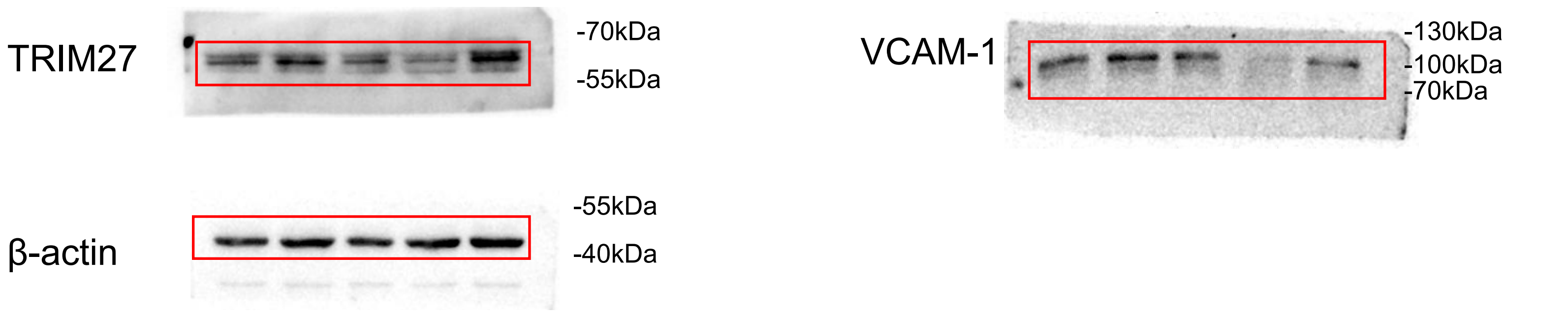

Figure 2d

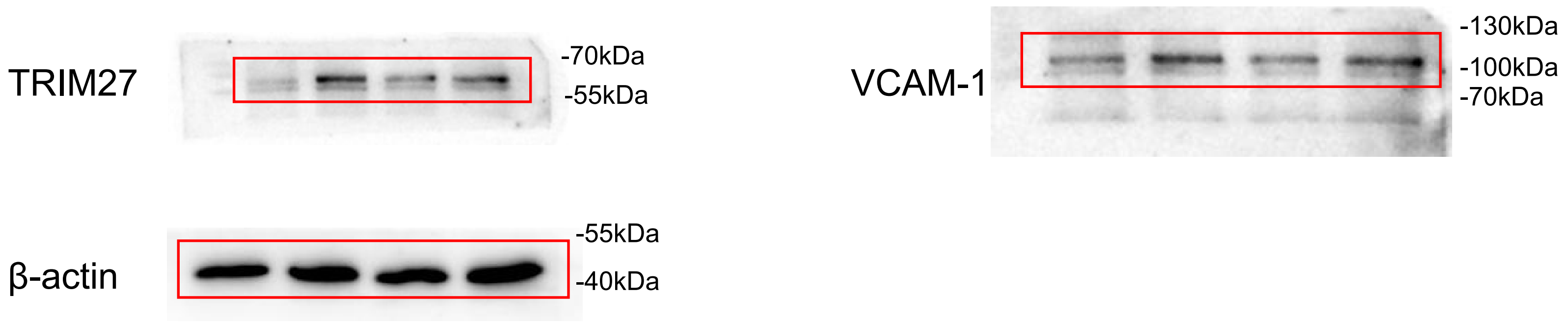

Figure 3a

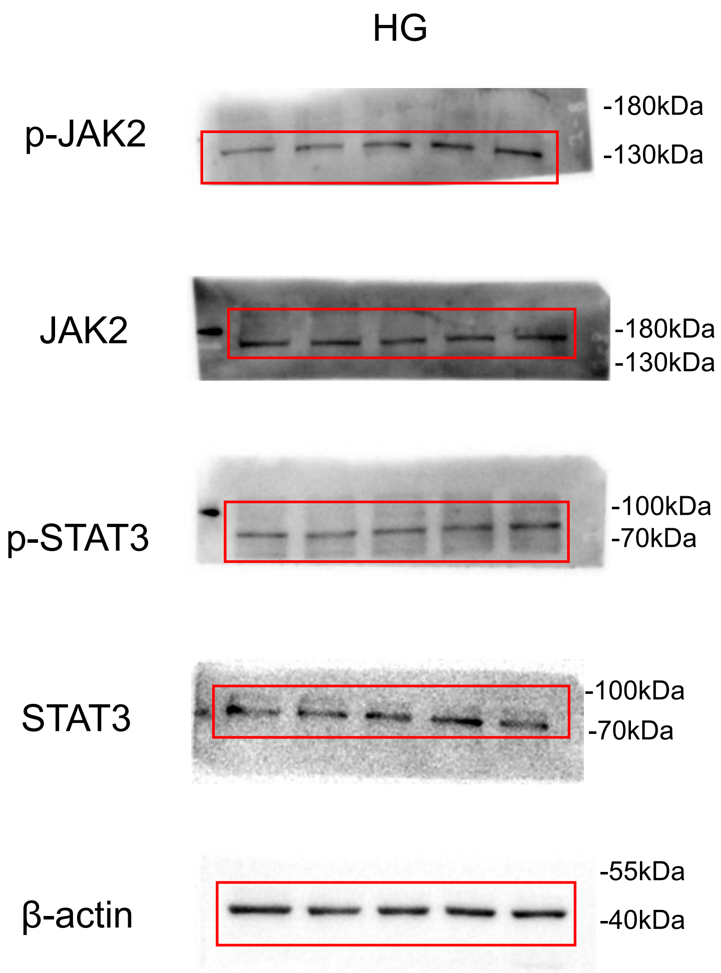

Figure 3c

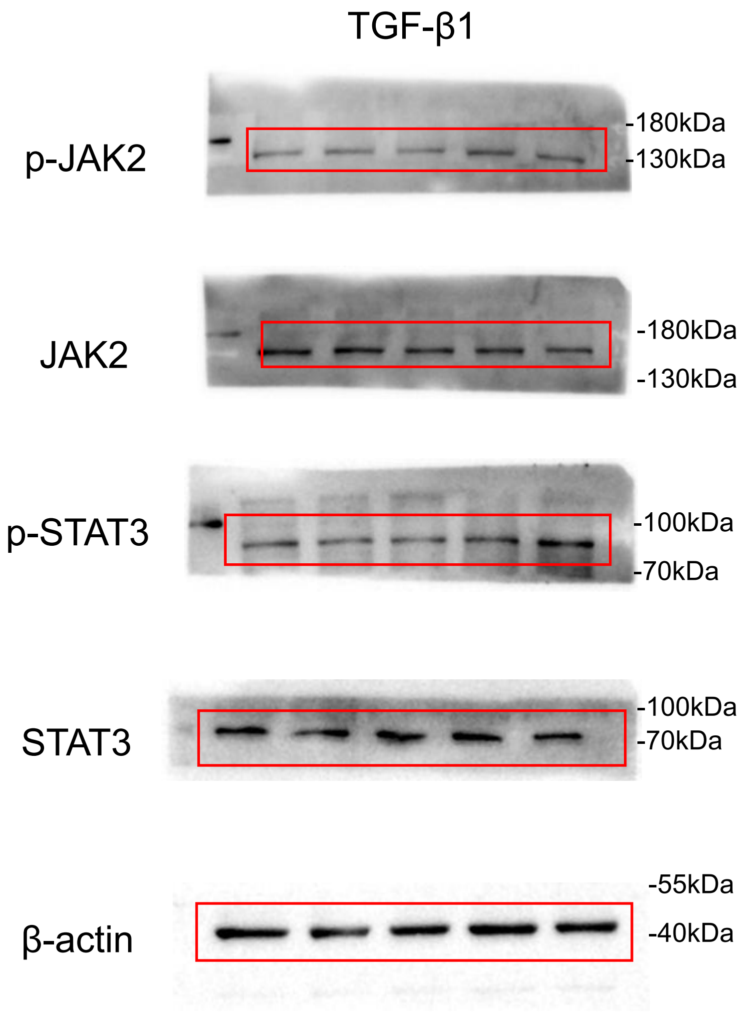

Figure 3e

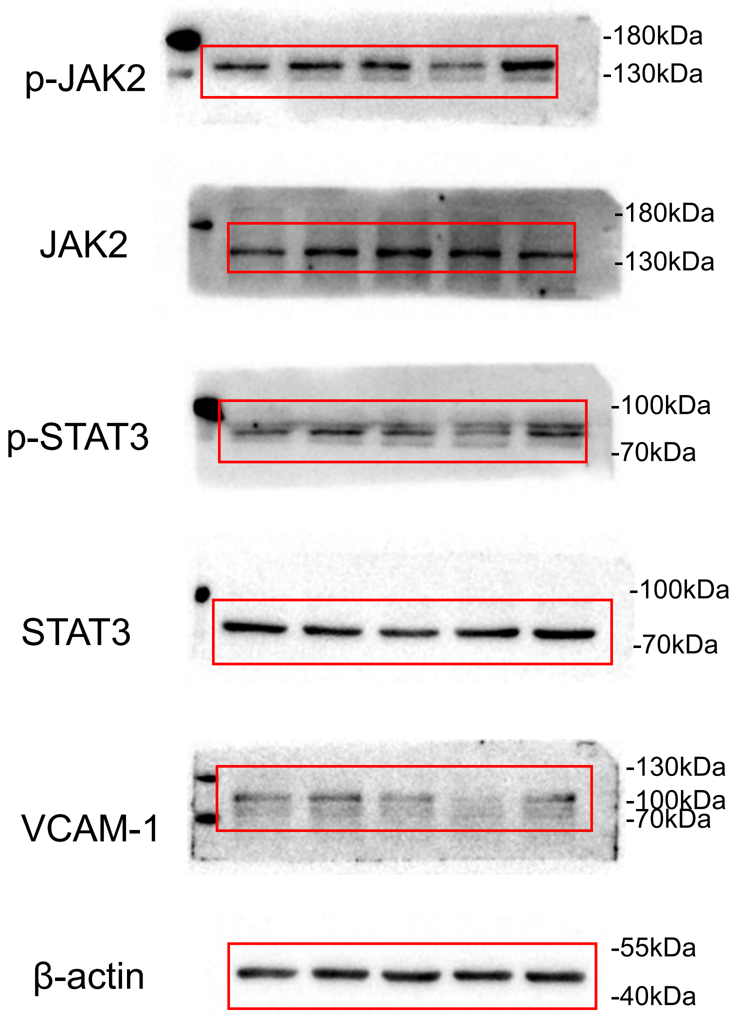

Figure 3l

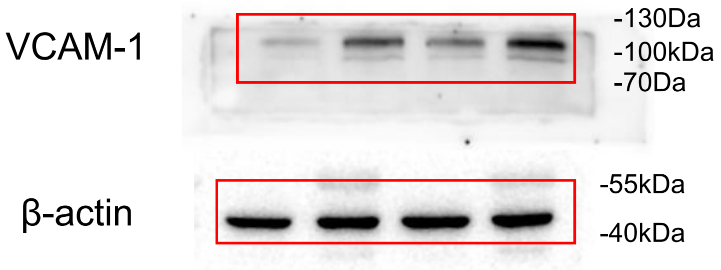

Figure 3l

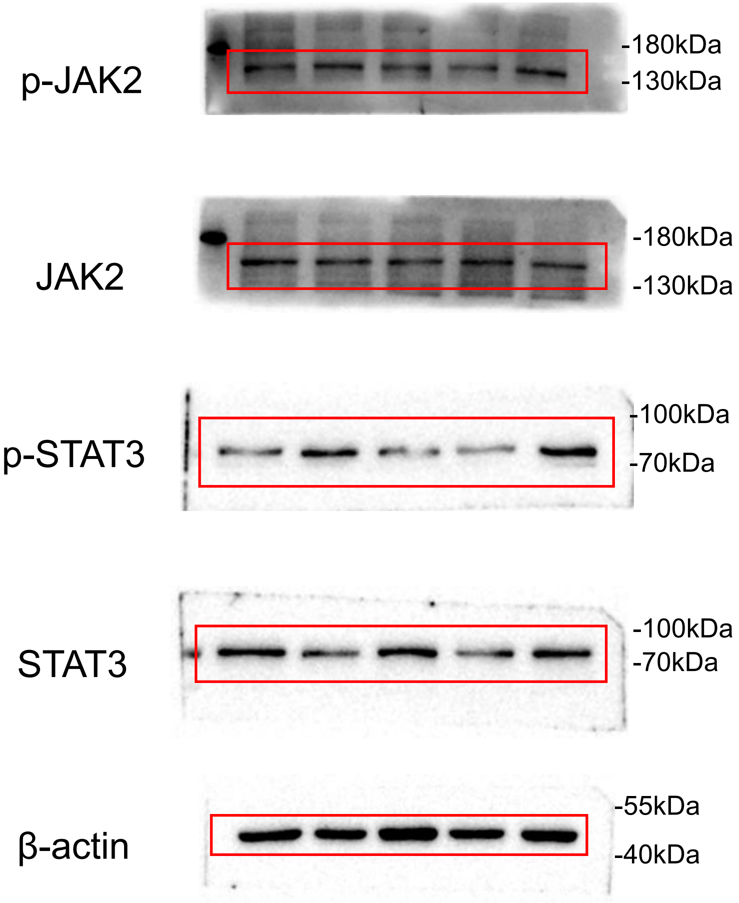

Figure 5b

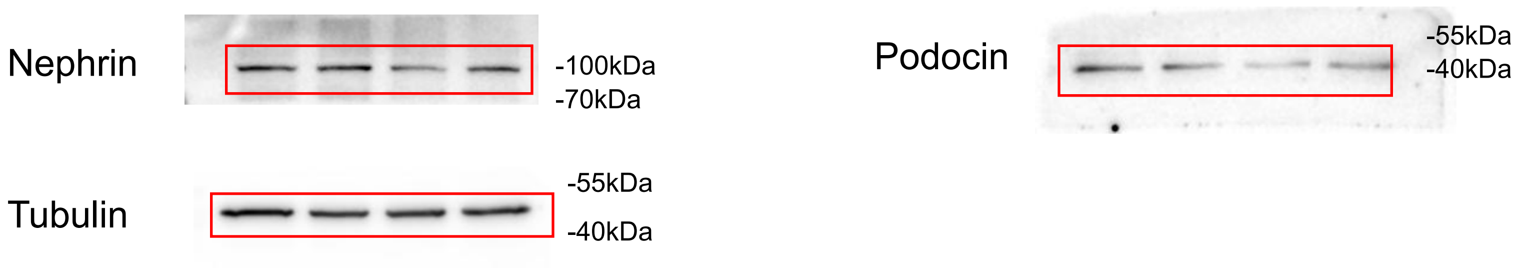

Figure 5d

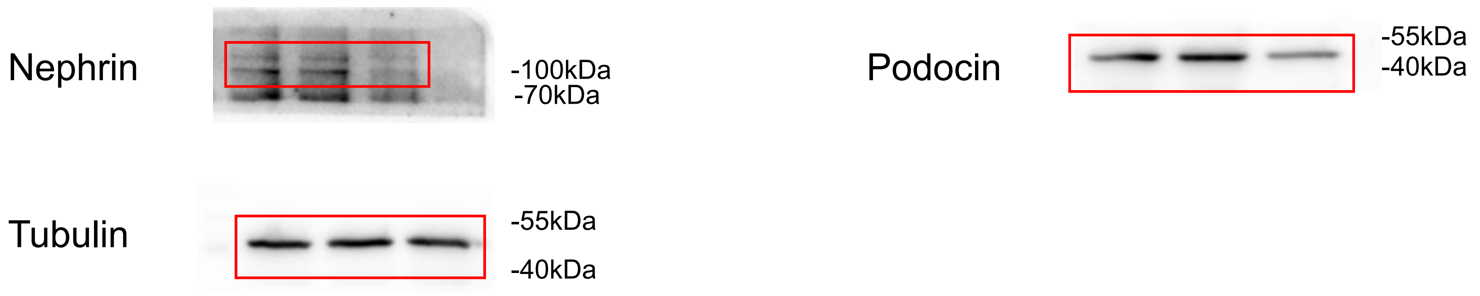

Figure 5f

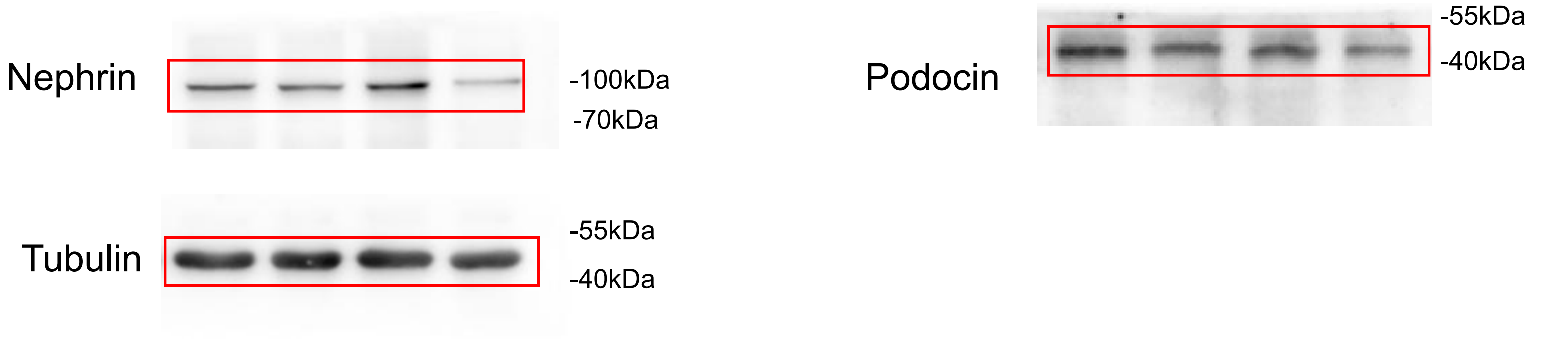

Figure 5k

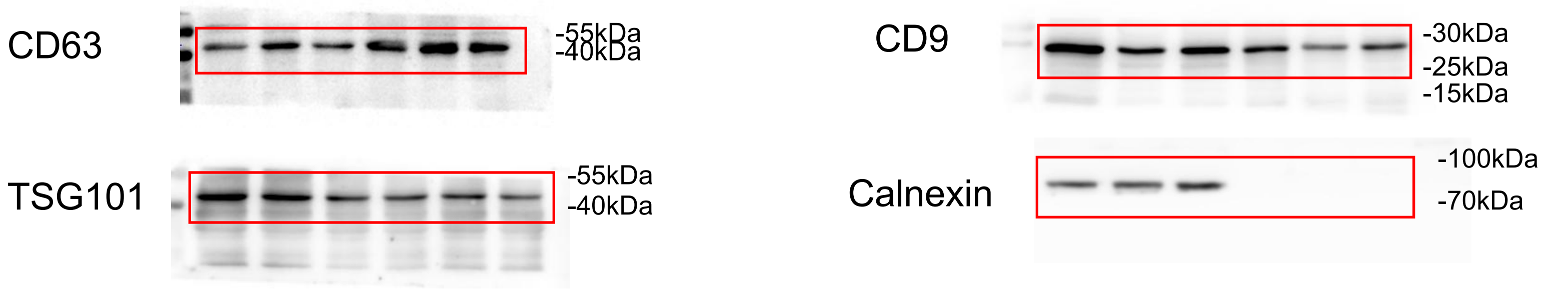

Figure 5n

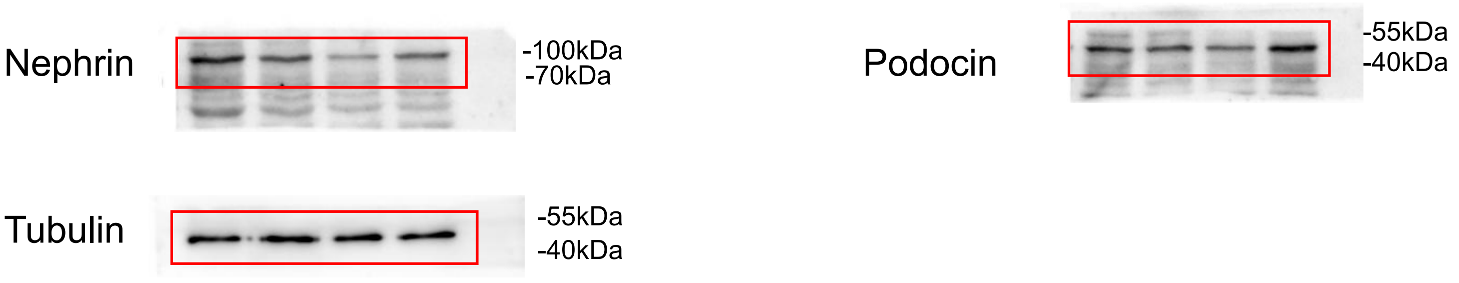

Figure 5p

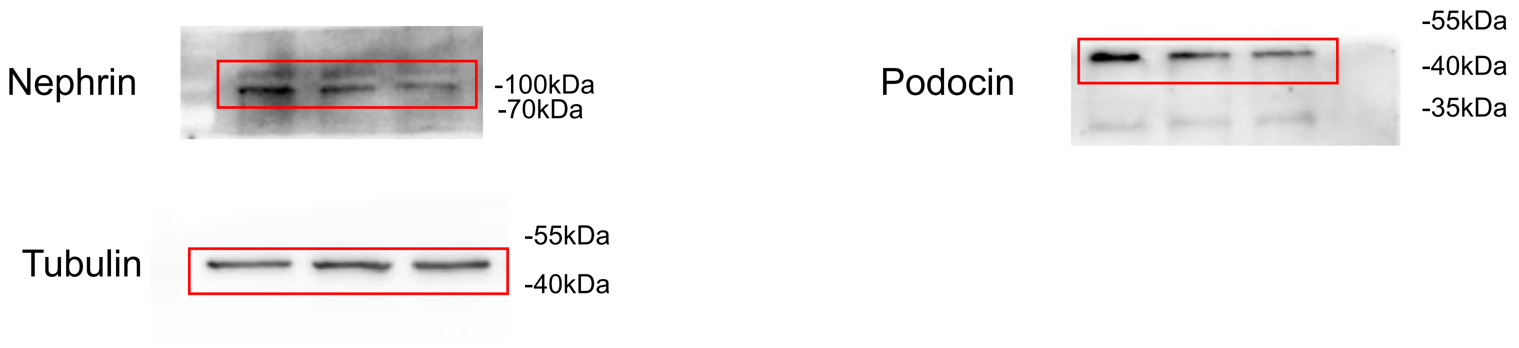

Figure 6d

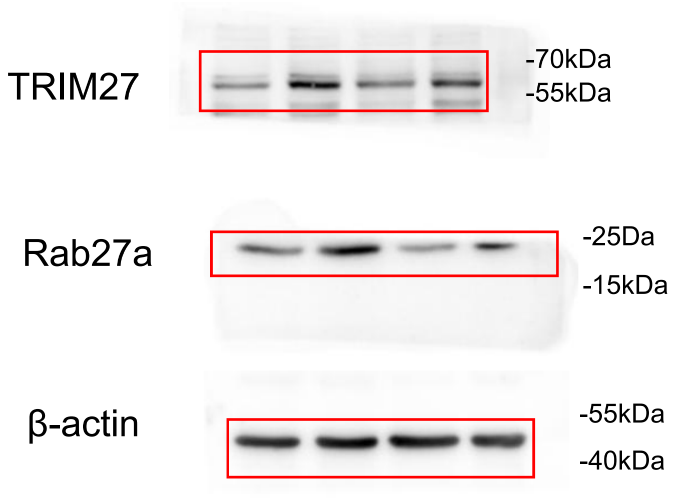

Figure 6h

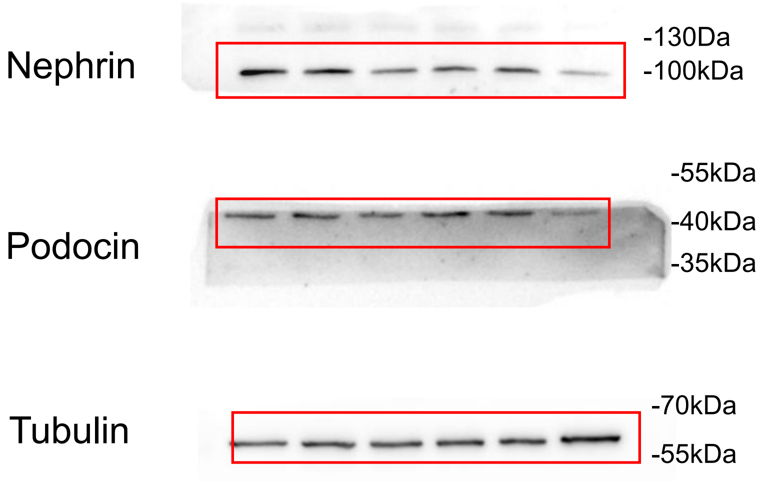

Figure 6f

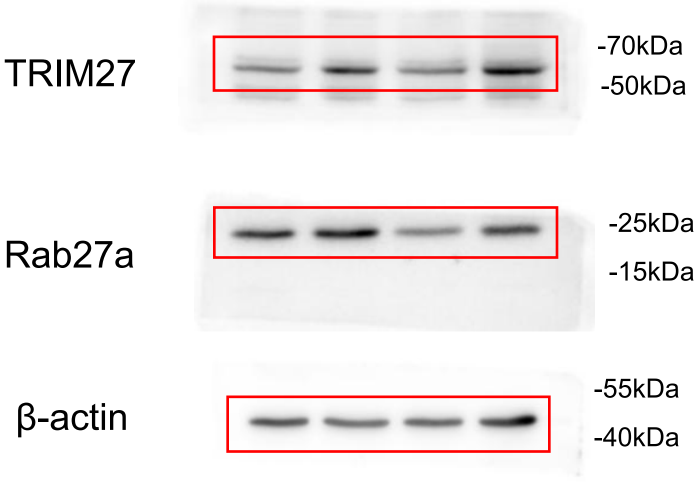

Figure 6j

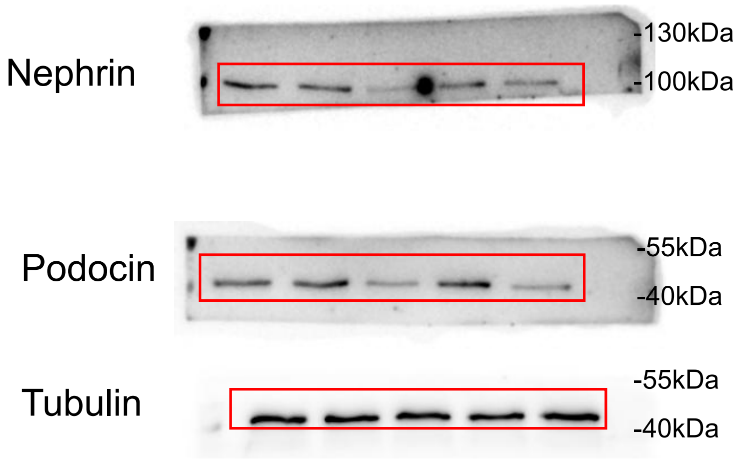

Figure 7g

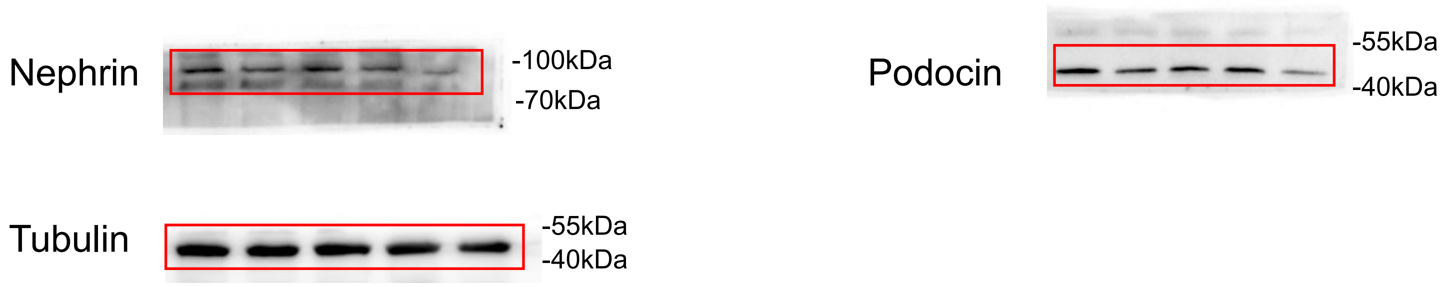

Figure 7i

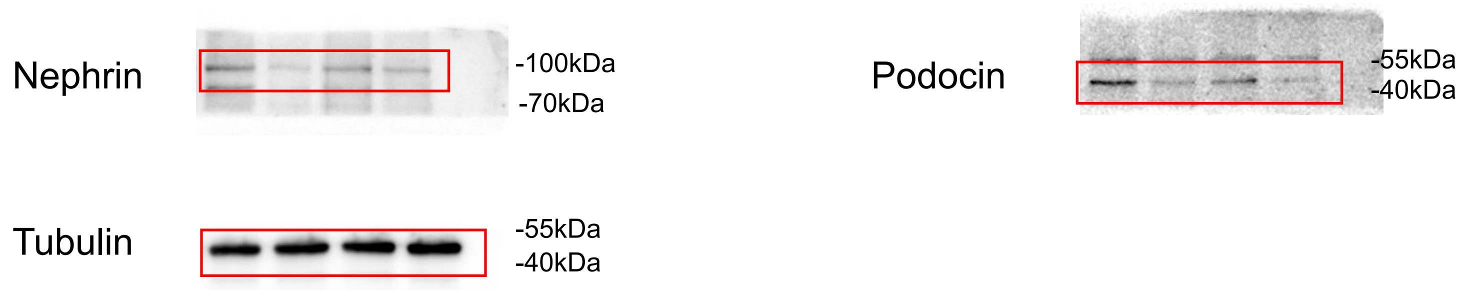

Figure 7n

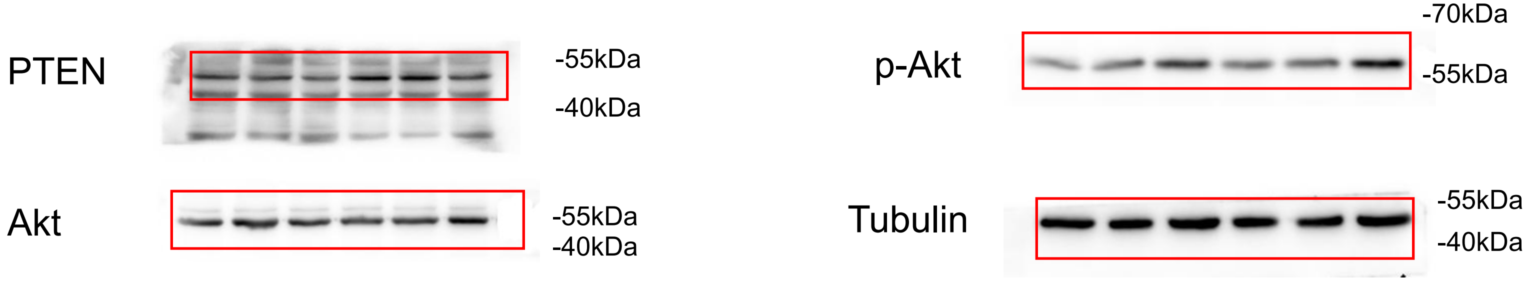

Figure 7p

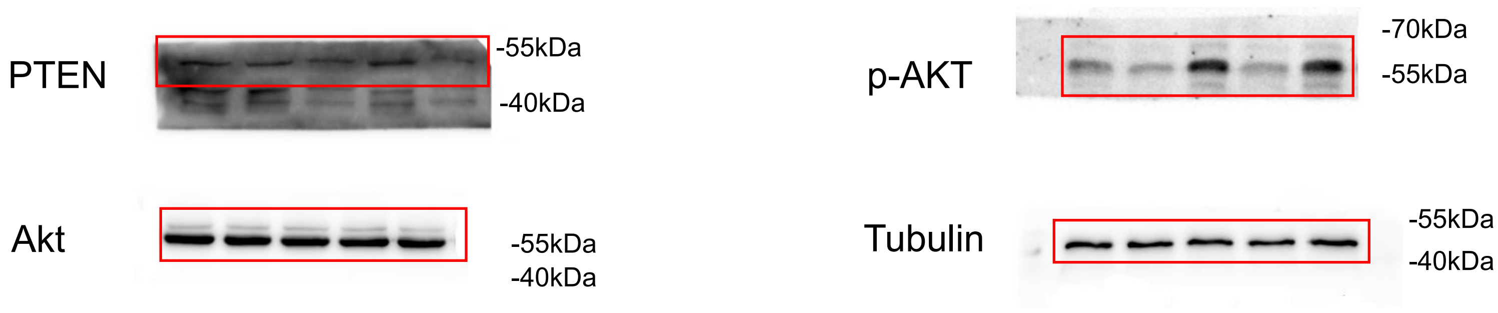

Figure 7r

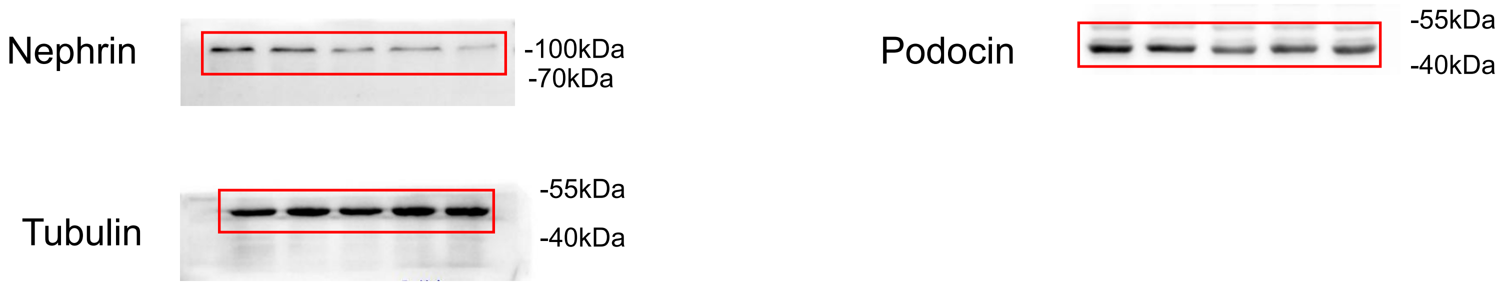

Supplementary Figure S2 e

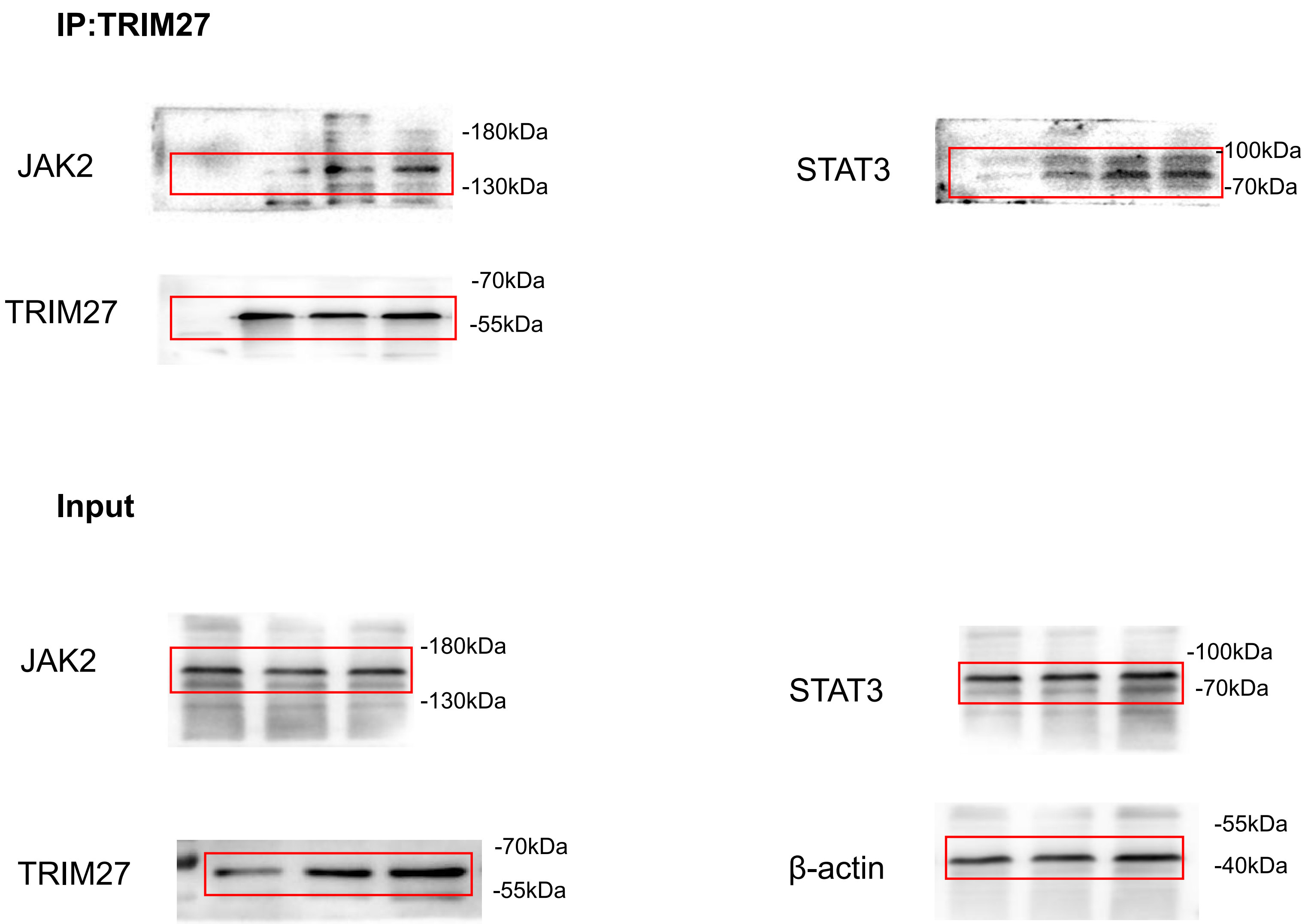

Supplementary Figure S5 a

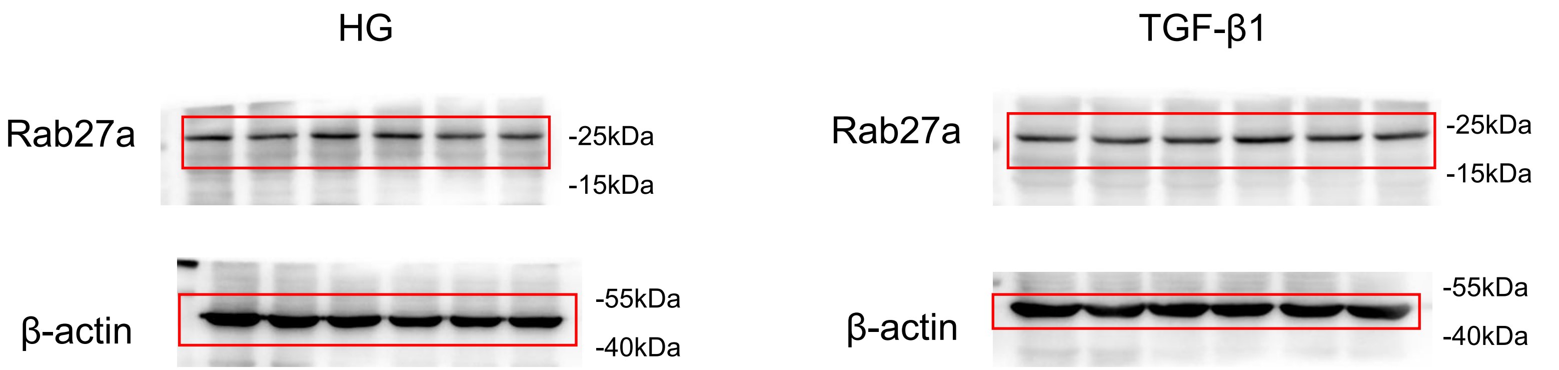

Supplementary Figure S5 g

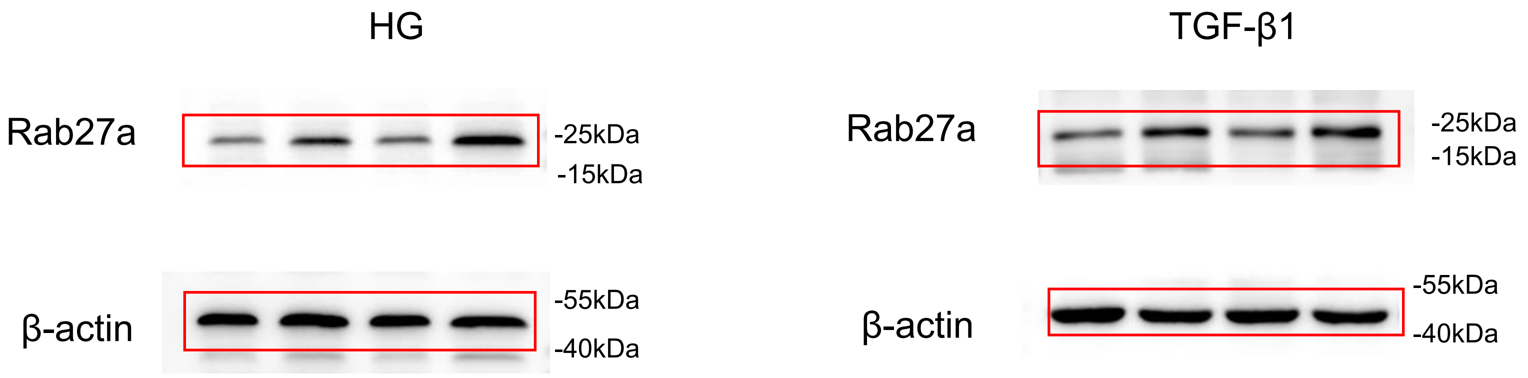

Supplementary Figure S6 C

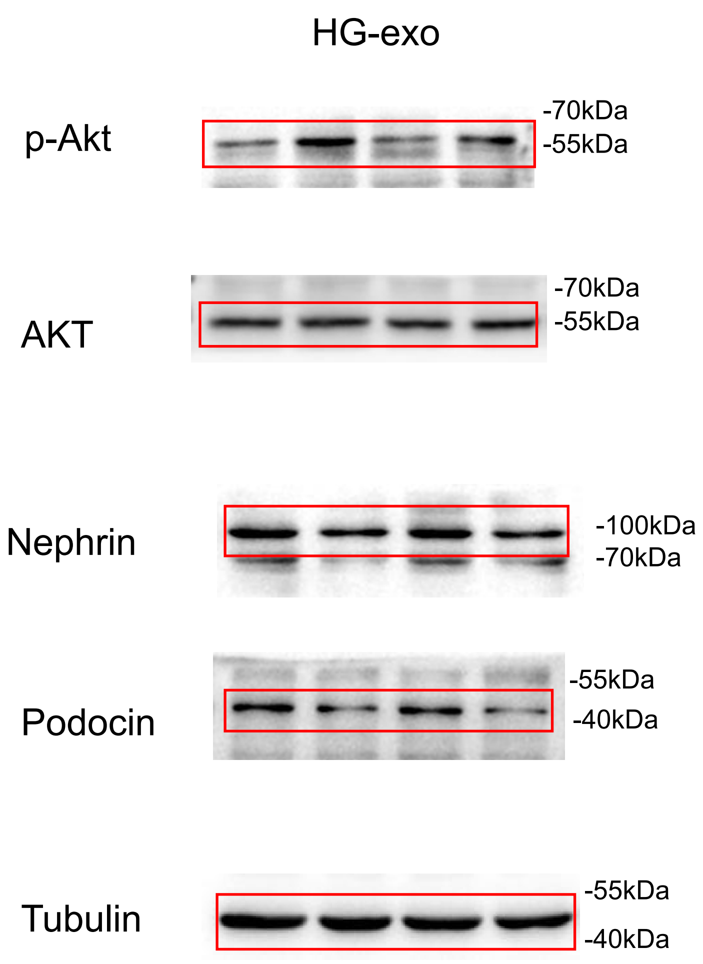

Supplementary Figure S6 D

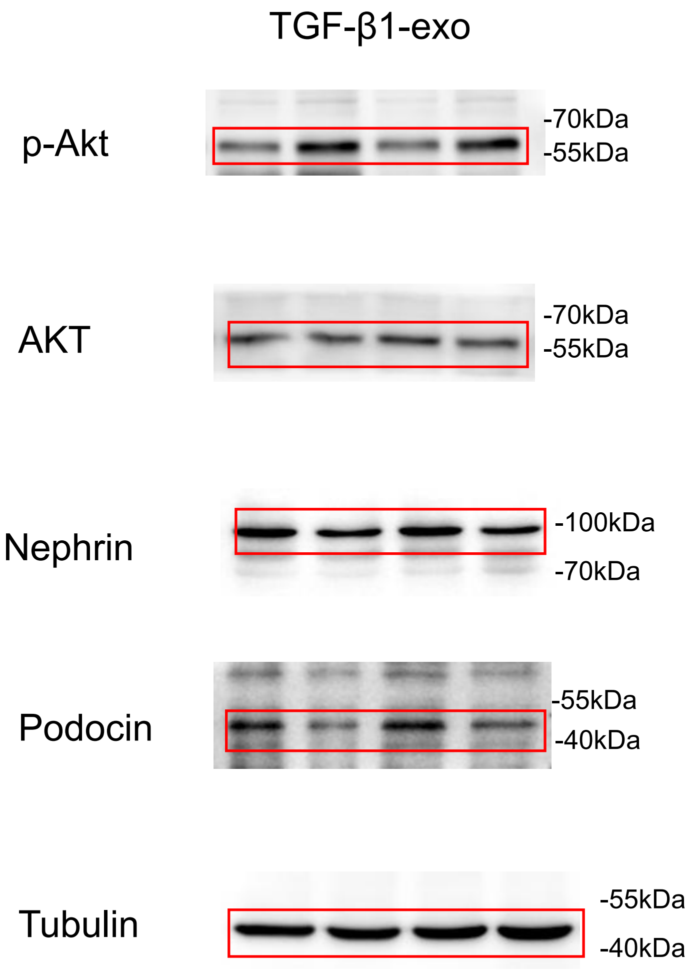

Supplement: Supplementary file 2 — Original Western blots [file 41420_2026_2953_MOESM2_ESM.pdf]
